# Advancement in continuous lactate determination in untreated human serum: multipolymer-based amperometric biosensor coupled with a low-cost 3D-printed microfluidic cell

**DOI:** 10.1007/s00604-025-07771-0

**Published:** 2025-12-27

**Authors:** Álvaro Jesús Sainz-Calvo, Alfonso Sierra-Padilla, Dolores Bellido-Milla, Lorena Blanco-Díaz, Juan Jesús Fernández-Alba, Carmen González-Macías, Juan José García-Guzmán, José María Palacios-Santander, Laura Cubillana-Aguilera

**Affiliations:** 1 Institute of Research on Electron Microscopy and Materials (IMEYMAT), Department of Analytical Chemistry, Faculty of Sciences, Campus de Excelencia Internacional del Mar (CEIMAR), University of Cadiz, Campus Universitario de Puerto Real, Polígono del Río San Pedro S/N, 11510, Puerto Real, Cádiz Spain; 2 Departamento de Obstetricia y Ginecología, Hospital Universitario de Puerto Real, Puerto Real, Cádiz, 11510 Spain

**Keywords:** Sonogel–carbon-based lactate biosensor, Amperometry, 3D printed microfluidic cell, Enzyme encapsulation, Untreated serum analysis, Real-time lactate monitoring

## Abstract

Enzyme saturation in biosensing has been established as a main hindrance in lactate monitoring due to the high concentration of this species in biological samples. In this paper, a Sonogel–carbon transducer has been used for the fabrication of an amperometric biosensor for lactate (LA) determination. The sensor was constructed by immobilizing lactate oxidase as the bioreceptor and coating the electrode surface with multipolymer layers to minimize enzyme saturation and improve operational stability. This biosensor shows adequate sensitivity (1.74 µA mM^− 1^ cm^-2^) in a linear working range from 0.2 mM to 20 mM, suitable for physiological lactate concentrations in biofluids. The response of the biosensor was studied in physiological medium using several potential interferents (glucose, uric acid, dopamine, ascorbic acid, KCl, NaCl, CaCl_2_ and BSA), demonstrating negligible interference from these common electroactive species. A microfluidic cell was designed and fabricated using polyethylene terephthalate glycol (PETG) through a rapid and low-cost 3D printing process. Finally, the prepared microfluidic cell was applied to lactate monitoring in flow regime using the biosensor with both synthetic and real untreated human serum samples, showing excellent correlation with two standard reference techniques and confirming its reliability. These results highlight the potential of the proposed microfluidic amperometric biosensor as a robust, real-time tool for biomedical lactate monitoring.

**Graphical Abstract:**

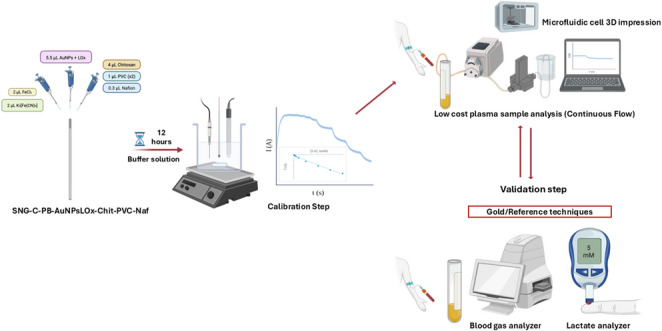

**Supplementary Information:**

The online version contains supplementary material available at 10.1007/s00604-025-07771-0.

## Introduction

The COVID-19 pandemic has underscored the inherent vulnerabilities and limitations in healthy systems, even in highly developed environments. Not only did this unprecedented global health crisis expose systemic weaknesses, but it also led to significant dissatisfaction and emotional exhaustion among healthcare professionals, largely due to the immense psychological and physical stress experienced during the pandemic [1, 2]. In light of these challenges, there is a growing interest in exploring innovative strategies to enhance diagnostic efficiency and reduce the burden on healthcare workers. One promising approach involves the use of specific metabolites-such as lactate, sodium, potassium and others-as potential biomarkers for the early detection and monitoring of various diseases like diabetes, cancer Alzheimer, Parkinson, heart attack, septic shock, among others [3, 4]. These biomedical indicators could serve as valuable tools in clinical settings, enabling timely diagnosis and improving patient outcomes while supporting healthcare providers in managing their workload more effectively [5].

Lactate is an important metabolite whose concentration in blood reflects metabolic activity and physiological status. Accurate, real-time monitoring of lactate gained interest in medicine and biomedical technology [6–9], as it enables continuous assessment of patients and athletes, supporting clinical decision-making and personalized interventions. Blood lactate levels vary with physiological conditions, exercise, or pathological states, highlighting its potential as a biomarker in both sports science and clinical diagnostics [10–12].

In healthy individuals at rest, blood concentrations of L-lactate typically range between 0.3 and 1.3 millimoles per liter (mM) [13]. However, during physical exertion, these levels can rise significantly, reaching approximately 12 mM under normal exercise conditions [14], and up to 15 mM in cases of extreme physical stress or high-intensity activity [15]. In particular, certain patients with mental disorders like epilepsy, Alzheimer, depression and schizophrenia may present markedly abnormal blood lactate concentrations [16]. These findings highlight the relevance of lactate as a potential biomarker, not only in sports science but also in medicine field.

Due to the clinical and technological importance of lactate monitoring, there is a growing need for fast, precise, cost-effective, and minimally invasive sensing methods. While traditional techniques such as colorimetric assays [17], nuclear magnetic resonance (NMR) [18] high pressure liquid chromatography (HPLC) [19] or gas chromatography coupled to mass spectrometry (GC-MS) [20], provide reliable measurements, they are unsuitable for point-of-care applications because of some drawbacks such as long analysis times, specialized workers and usage of expensive instrumentation and reagents.

In response to these challenges, electrochemical biosensors, particularly those employing an enzyme as a bioreceptor, have emerged as a promising alternative to conventional methods for lactate quantifications in medical applications. Electrochemical sensing relies on the measurement of electrical signals generated by the interaction between a target analyte and a transducer. In enzymatic biosensors, the enzyme catalyzes a specific reaction with the analyte, producing electroactive species, like H_2_O_2_, which can be detected using several electroanalytical techniques. These devices are capable of overcoming many of the limitations previously described, owing to the unique combination of the high sensitivity and specificity of biological recognition elements with the efficiency of physicochemical transducers, enabling complex analytical measurements through simple, user-friendly devices [21]. Consequently, these biosensors offer several significant advantages, including low manufacturing cost, small dimensions for easy in situ measurements, minimal sample preparation requirements and portability [22]. In particular, the integration of electrochemical lactate biosensors into microfluidic platforms has gained attention due to its potential to enhance performance by allowing precise control over sample volumes, improving reaction kinetics, and facilitating automation and continuous monitoring [23]. Microfluidic systems are especially attractive for point-of-care diagnostics, as they require low reagent volumes, support device miniaturization, and can be manufactured using cost-effective techniques such as screen-printing, inkjet printing, and photolithography [24]. The integration of these biosensors leverages several advanced techniques to further improve sensor functionality in real-world conditions. Precise microchannel designs incorporating bubble-trapping structures mitigate flow disruptions, which is critical for continuous monitoring in complex fluids such as sweat [25]. Besides, automation within microfluidic platforms, enabled by components such as peristaltic pumps and electrovalves, facilitates real-time monitoring of metabolites in cell culture models over extended periods without extensive sample preparation. The use of spin-coated multilayer films, including biocompatible polymers like chitosan and Nafion [26], enhances sensor stability and selectivity when analyzing complex biological matrices such as plasma and serum under continuous flow conditions. Additionally, multiplexed microfabricated chips featuring hydrophobic barriers allow simultaneous detection of multiple analytes with enhanced signal amplification using stop-flow techniques [27]. Collectively, these integration strategies address critical challenges such as sample volume control, matrix interference, and continuous operation, thereby advancing the practical deployment of electrochemical lactate sensors in clinical and sports monitoring applications [28].

However, despite these clear advantages, only a limited number of electrochemical lactate sensors have been tested under continuous flow conditions or evaluated using complex biological matrices such as plasma or serum [26]. This represents a critical gap in the current research landscape, as validating these devices under real-world conditions is essential for ensuring reliable performance in clinical environments, where factors such as matrix interference and sample variability can significantly impact sensor accuracy and robustness.

Most biosensors use a specific enzyme immobilized on a transducer, which converts physical or chemical changes into measurable signals. In lactate biosensors, lactate oxidase is commonly applied to catalyze the oxidation of L-lactate to pyruvate, with the simultaneous reduction of O_2_ to H_2_O_2_. Since oxygen acts as an electron acceptor, both O_2_ and the related product H_2_O_2_ can be electrochemically monitored, generating an amperometric output that is proportional to the L-lactate concentration.

In this regard, in traditional techniques employing lactate oxidase, a platinum electrode was commonly used to detect H_2_O_2_. However, platinum exhibits low selectivity for H_2_O_2_, requiring high anodic potential that can lead to the detection of other interfering species with strong oxidizing properties [29]. In recent years, ferric ferrocyanide, commonly known as Prussian Blue, has been introduced as redox intermediate due to its high selectivity for the oxidation of hydrogen peroxide at low potentials, effectively minimizing interference. Even so, Prussian Blue has a drawback, and that is its saturation at high lactate concentration, so in several references [24, 25] polymeric membranes such as chitosan, polyvinyl chloride and Nafion™ have been used to limit the access of lactate and other interferents to the enzyme and, thus, to optimize its functioning [30].

Last but not least, as previously discussed, most biosensors should be capable of analyzing samples eliminating the requirement for pre-treatment and supporting continuous operation. Biosensors are crucial in diagnostics and prognostics, offering deeper insight and trend predictions in analyte concentration than other techniques. This application in continuous regime has, in addition to the advantage of real-time monitoring of the sample, the optimization of required sample volume. This is possible through the use of microfluidic cells that minimize the volume required, which is of particular interest when working with physiological fluids. Notably, biosensors based on sonogel materials have demonstrated excellent performance in the analysis of diverse samples [31–34], including biomedical [35, 36], showing high sensitivity, stability, and reproducibility, which makes them particularly suitable for integration in microfluidic platforms. While previous studies have demonstrated the reliability of sonogel-based biosensors [34] and explored the use of low-cost 3D printing for microfluidic device fabrication [37, 38], this work integrates both elements to develop a continuous lactate monitoring system combining high sensor stability with a fully customized 3D-printed microfluidic cell tailored for complex biological samples such as plasma and serum. Unlike prior approaches, our design enables real-time analysis under continuous flow conditions with minimal sample volume and without pre-treatment, addressing a critical gap in current biosensor applications. Not only does this integration advance practical point-of-care diagnostics, but also paves the way for scalable, cost-effective manufacturing of wearable and portable lactate monitoring devices. Currently, there are no microfluidic cells for the type of biosensor to be developed in this work, so it is necessary to design and manufacture them. For this reason, 3D impression would be the best option to be applied in this paper to produce a microfluidic cell to detect lactate in continuous regime.

In the present work, to prevent enzyme saturation, a multipolymer-based amperometric sensor is developed with the aim to work in the range of 0.2–20 mM of lactate, in order to cover the common/uncommon range in blood. Moreover, its performance is compared with two reference methods: one typically applied in hospitals and the other commonly used at home by people suffering from some kind of lactate-related disease or by sport medicine purposes. Importantly, this work introduces a novel and cost-effective flow analysis platform for lactate determination by integrating two key strategies. First, a reliable biosensor is fabricated using a simple drop-casting method to create a multipolymer layer, ensuring its efficiency. Second, a microfluidic cell, designed for optimal flow conditions, is produced via low-cost 3D printing. While similar strategies have been previously reported, the novelty of this study lies in their integration into a compact platform capable of performing real-time lactate monitoring in untreated serum human samples under continuous flow conditions. This ability to operate without sample pretreatment represents a significant practical advantage, enhancing the simplicity, speed, and applicability of lactate determination in real-world biomedical scenarios. In addition, the platform designed emphasizes robustness and reproducibility, which are essential for its translation to clinical and point-of-care contexts. Finally, and more importantly, the validation of this platform was also successfully achieved by two different means, demonstrating its reliability and potential for routine use in biomedical and clinical practice.

## Materials and methods

### Reagents

All the reagents used were employed without further purification. Potassium hexacyanoferrate (III), potassium tetrachloroaurate (III), chitosan of medium molecular weight, polyvinyl chloride, tetrahydrofuran, Nafion™ in perfluorate resin solution, uric acid, lactate (LA), dopamine hydrochloride, bovine serum albumin (BSA), lactate oxidase (LOx) from *Aerococcus viridans* (40 enzymatic units per mg), hemoglobin from bovine blood and bilirubin were obtained from Sigma-Aldrich (Burlington, USA). Acetylsalicylic acid was purchased from Labkem (Ireland). The acetaminophen was obtained from DropSens (Llanera, Spain). Methyltrimethoxysilane (MTMOS), hydrochloric acid, sulfuric acid, acetic acid and potassium chloride were purchased from Merck (Darmstadt, Germany). Dipotassium hydrogen phosphate, potassium dihydrogen phosphate, potassium hydroxide, sodium hydroxide, iron (III) chloride hexahydrate, ethanol, ammonium, hydrogen peroxide, D(+)-glucose anhydrous and calcium chloride were procured from Panreac (Barcelona, Spain). Graphite powder from Alfa Aesar (Germany, Johnson Matthey GmbH) was used for carbon ceramic electrodes fabrication, whereas tribasic sodium citrate hydrate from Riedel-de Haën (Seelze, Germany) was used for gold nanoparticles synthesis. Nanopure water was obtained from a Wasser Lab Ultramatic Plus (Type I) system (18 MΩ cm, Barbatáin, Navarra, Spain). (Glass capillary tubes, i.d. 1.15 mm, were used as the bodies of the composite electrodes. polyethylene terephthalate glycol (PETG) filament was obtained from Prusa (Praga, Chez Republic).

### Instrumentation

The high-power ultrasound-assisted synthesis of the electrode materials was performed using a MISONIX S-4000 ultrasonic generator (USA, ME, Farmingdale) coupled with a 13 mm diameter titanium tip. The electrochemical measurements were made using an Autolab PGTSTAT12 potentiostat/galvanostat coupled to a 663 VA stand from Metrohm (The Netherlands, Utrecht). The software GPES (General Purpose Electrochemical System) was used for data acquisition and elaboration. A three-electrode cell with the following composition was employed: a platinum wire as a counter electrode, a Ag/AgCl (3 M KCl) as the reference electrode and the Sonogel–Carbon-based composites as the working electrodes.

UV–visible measurements were made using a Jasco V-550 (Easton, MD, USA) UV-visible spectrophotometer. Size distribution analysis with dynamic light scattering (DLS) technique was performed with a Microtrac Nanotrac Wave (Duesseldorf, Germany) particle analyzer equipped with a laser diode emitting at a wavelength of 780 nm, with a nominal power of 3 mW.

Scanning electron microscopy (SEM, [FEI Nova NANOSEM 450], Thermo Fisher Scientific, USA) micrographs were taken at 5–20 kV using secondary electron detector at different magnifications. This microscope is also coupled to an EDAX unit to perform X-ray energy dispersive spectroscopy (EDS).

The AFM images were obtained using a Dimension Icon microscope (Bruker, Massachusetts, USA) operating in Peak Force Tapping mode using ScanAsyst-Air probes (stiffness 0.2–0.8 N/m, frequency∼80 kHz).

FT-IR spectroscopy was carried out using a Perkin Elmer Spectrum BX spectrophotometer (Waltham, MA, USA), in the attenuated total reflectance (ATR) mode in the wavenumbers interval ranging from 900 to 4000 cm-1.

FreeCAD software 0.20 was used for the design of a 3D microfluidic cell, whereas PrusaSlicer 26.1 software was used to slice the design to printing data. The impression of this cell was performed with a 3D printer Original Prusa MINI+ (Prusa, Czech Republic) using polyethylene terephthalate glycol (PETG) as polymeric filament. A peristaltic pump Minipuls 2 from Gilson (Wisconsin, United States) was used for the flow regime measurements. A GEM Premier 4000 gas analyzer and a Lactate Scout analyzer 4 (Barleben, Germany) were used for the determination of lactate in real samples for comparing purposes.

The electrochemical impedance data were analyzed using ZView software (version 3.5, Scribner Associates, USA). The program was employed to fit the experimental impedance spectra and calculate the real (Z’) and imaginary (Z’’) components, allowing the determination of the chi-squared (χ²) values for obtaining the goodness of fit.

### Preparation and conditioning of Sonogel-Carbon electrodes

The Sonogel-Carbon (SNG-C) electrodes were prepared as described in a previous report [39]. In brief, a reaction mixture consisting of 100 µL of 0.2 M hydrochloric acid solution and 500 µL of MTMOS precursor was sonicated for 10 s at 40% amplitude using a high-power ultrasound probe. Then, 500 mg of graphite of spectroscopic quality was added and uniformly mixed to obtain a paste. Following this, the electrodes were fabricated by filling capillary tubes with the prepared material. After a mild surface polishing with P1200 emery paper (Struers, Germany) and insertion of a copper wire to establish electrical contact, the electrodes (with a geometric area of 1.04 × 10^− 2^ cm^2^) were deemed suitable for use.

Before their use, the operational SNG-C electrodes surface underwent electrochemical conditioning by means of cyclic voltammetry in free analyte phosphate buffer solution by recording five cyclic sweeps from − 0.6 V to 0.8 V and vice versa at 50 mV/s. Afterwards, these electrodes were electrochemically activated in a 0.1 M H_2_SO_4_ aqueous solution via two polarization stages at − 0.7 V for 10 s and at + 1.8 V for 10 s. This electrochemical procedure was repeated four times [40].

### Synthesis of gold sononanoparticles

Gold sononanoparticles (AuSNPs) were synthesized using a previously described method [8]. Firstly, a cylindrical glass vessel was prepared with 1.25 mL of 1.5 mM KAuCl_4_ aqueous solution, which was sonicated with a high-power ultrasound probe set at 13% amplitude for 1.5 min. Then, 250 µL of 38.8 mM sodium citrate aqueous solution was added to the vessel. It took 4 min for the solution to change colour to dark red wine, indicating the formation of AuSNPs. The sample vessel was kept in a water bath for the entire process to prevent any localized heating caused by sonication. The obtained AuSNPs were characterized using UV-Vis spectrophotometry and dynamic light scattering techniques.

### Fabrication of the lactate biosensor

The lactate biosensor, named as Sonogel-Carbon-PrussianBlue-LactateOxidase-GoldSononanoparticles-Chitosan-PolyvinylChloride-Nafion (SNG-C-PB-LOx-AuSNPs-Chit-PVC-Nafion) was fabricated using a layer-by-layer modification procedure. Initially, a mediator layer comprising Prussian Blue (PB) was drop-casted onto the surface of a Sonogel-Carbon electrode. This was done by blending 2 µL of a 0.1 M KCl, 0.1 M FeCl_3_ and 0.01 M HCl solution with 2 µL of a 0.1 M KCl, 0.1 M K_3_Fe(CN)_6_ and 0.01 M HCl solution. The mixture was removed after 20 min, followed by rinsing of the electrode with 4 µL of 0.01 M HCl and 4 µL of nanopure grade water. The electrode was then annealed in an oven at 100 °C for 1 h.

Next, the enzyme layer was created by dispersing 5.5 µL of a solution containing 2.4 mg of lactate oxidase (100 U) in 260 µL of AuSNPs solution, where gold nanoparticles catalyze and enhance the enzyme activity [41]. This coating was left to air dry for approximately 3 h. Subsequently, the diffusion membrane was constructed by assembling multiple polymeric layers. The initial layer was made by dispersing 4 µL of a 1% w/v chitosan in 0.1 M acetic acid (adjusted at pH 4.5) mixture and drying it for 2 h, this first layer is used to immobilize the enzyme on the electrode surface. Then, 1 µL of a solution with a PVC concentration of 10 mg/mL in tetrahydrofuran was dispersed twice, allowing it to dry in a few minutes each time. Finally, 0.5 µL of a 1% Nafion solution in ethanol (adjusted at pH 7.5) was cast by droplet and left to dry for 1 h; these outer layers are used to control the access of lactate to the enzyme, thereby preventing enzyme saturation. The drying steps were carried out in darkness at room temperature. The biosensors were stored in 0.1 PBS at pH 7.4 in darkness and at 4 °C after their first use in electroanalytical applications.

### Morphological, structural, spectroscopic and electrochemical study of the redox mediator and polymeric layers of the biosensor

To understand how sequential layer deposition affects the lactate biosensor performance, a multi-technique characterization approach was employed, correlating structural, chemical, and electrochemical properties. The surface morphology of unmodified and Prussian Blue (PB)-modified SNG-C electrodes was examined using scanning electronic microscopy (SEM). This analysis provided information on layer uniformity, porosity, roughness, and mediator distribution, which are critical for electron transfer efficiency and enzyme accessibility. In addition, atomic force microscopy (AFM) was performed to obtain high-resolution topographical information, enabling quantitative analysis of surface roughness and nanoscale features that complement the SEM observations.

EIS measurements were performed at each modification stage, including the redox mediator, enzymatic mixture, and polymeric layers. Key parameters such as charge transfer resistance (Rp) and solution resistance (Rs) were extracted to evaluate the effects of each layer on electron transfer kinetics, mediator accessibility, interference reduction, and enzyme stabilization. The study was conducted using a Frequency Response Analyzer (FRA). The biosensor was immersed in 25 mL of a solution containing 5 mM K₄[Fe(CN)₆] and K₃[Fe(CN)₆], with 0.5 M KNO₃ or KCl as the supporting electrolyte. The frequency range was set from 0.1 Hz to 10 kHz, with 50 frequency points. An amplitude of 5 mV and a potential of 0.25 V were applied during measurements [41].

### Analytical performance of the biosensor in batch mode

The electrochemical performance of the device was assessed in an electrochemical cell using the lactate biosensor as the working electrode, an Ag/AgCl/KCl 3 M electrode as the reference electrode, and a platinum electrode as the auxiliary electrode. Chronoamperometry technique was employed under a potential of 0.10 V (vs. Ag/AgCl). Additionally, a buffer solution consisting of 0.1 M K_2_HPO_4_ and 0.1 M KH_2_PO_4_ (PBS) with an adjusted pH of 7.4 was selected due to its similarity to physiological conditions. The biosensor was calibrated in the presence of lactate, using concentrations ranging from 0.2 to 20 mM. Adequate volumes were added from 1000 mM to 100 mM lactate stock solutions in PBS. From these data, the analytical parameters of the biosensor–such as sensitivity, limit of detection (LOD), limit of quantification (LOQ), and linearity–were calculated using the calibration curve according to standard IUPAC criteria.

To further evaluate the enzymatic behavior of the lactate biosensor, Michaelis-Menten kinetic study was carried out under the same chronoamperometric conditions described above. The steady-state current responses were recorded at increasing lactate concentrations and plotted as a function of substrate concentration to obtain the characteristic saturation curve. The apparent Michaelis constant (K_m_) and maximum current (I_max_) were determined by fitting the experimental data to Michaelis-Menten equations (Eq. 1) for enzyme-based electrochemical systems. This analysis makes it possible to assess the affinity of the immobilized enzyme for lactate and to characterize the catalytic efficiency of the biosensor, providing deeper insight into its operational performance.

1 $$\mathrm I=\frac{\mathrm{Imax}\cdot\left[\mathrm S\right]}{\mathrm{Km}+\left[\mathrm S\right]}$$

Reproducibility of the biosensor was assessed by calibration of lactate with three different electrodes, whereas repeatability was evaluated by performing the calibrating with a biosensor by triplicate. The lifetime of the biosensor was evaluated by recording their signal in a 2 mM lactate solution for 1, 2, 6, 13, 19 and 21 consecutive days. In addition, the stability of the biosensor was assessed in real human serum at different lactate concentrations using the same biosensor over several days, analyzing one sample (in triplicate) per day. Signal stability was recorded in buffer solution for 8 h. The study was carried out in 0.1 M PBS and artificial serum with the following composition: bovine serum albumin (BSA) 0.1%, NaCl 105 mM, NaHCO_3_ 26 mM, KCl 4 mM, NaH_2_PO_4_ 1.7 mM, CaCl_2_ 1.2 mM, glucose 4.7 mM and urea 2.5 mM [42], at both low (1 mM) and high (15 mM) lactate concentrations.

The selectivity of the developed device was studied using several species usually found in physiological media and at biological concentrations. The calibration of the biosensor with lactate was carried out in a media containing 264 µM paracetamol, 4.33 mM bilirubin, 41 µM salicylate, 0.05 mg/mL hemoglobin, 100 µM ascorbic acid, 100 µM uric acid, 6 µM dopamine, 5 mM glucose, 20 mg/mL BSA, 3.5 mM KCl, 140 mM NaCl and 1.5 mM CaCl_2_ in 0.1 M PBS at pH 7.4 [43–45].

### Manufacturing of a microfluidic cell

Several microfluidic cells were designed (FreeCAD ver 0.20) and fabricated with a low-cost 3D printer (Prusa Mini, Prusa Research Czech Republic) for its application with the lactate biosensor in continuous analysis mode. The cells were made of PETG filament of 1.75 mm diameter and are composed by four pieces: the lower part, the upper part and two screws. Different fabrication parameters of layer distance and filling percentage were used for each piece: 0.10 mm and 80% for the base; 0.15 mm and 60% for the casing; and 0.07 mm and 15% for the screws. A nozzle of 0.4 mm was used with 230 °C and 240 °C of temperature for the first layer and the successive ones, respectively, whereas the stand temperature was 75 °C and 80 °C, respectively as well. The dimensions of the microfluidic cells are specified in Figures S1, S2, S3 and S4 of the Supplementary Material. The diameter and length of the inner channel are 2 and 30 mm, respectively, whereas the total volume of the microfluidic cell is about 280 µL. Moreover, the dimensions of the upper and lower sections of several (*n* = 4) microfluidic cells are reported in Table S1 for reproducibility purposes.

In the central part of the lower section, three holes of 2 mm diameter were included to insert the lactate biosensor, the reference electrode, and the auxiliary electrode, ensuring direct contact with the sample. A channel was integrated into the base to allow the sample flow through the cell. Finally, two screws located at the ends of the cell hold thin plastic tubes that are connected to a peristatic pump, which drives the sample into the cell, to reach the electrodes, and out through the opposite side. The upper part of the cell was mainly designed to provide mechanical support for the electrodes and to facilitate their connection to the potentiostat, without playing any role in sample flow. Detailed blueprints are exposed in Supplementary material (Figures S1-S2).

### Analysis of lactate in continuous mode

The 3D printed microfluidic cell was used for the determination of lactate with the prepared biosensor. The electrochemical cell for this purpose was constituted by the lactate biosensor as the working electrode, a bare SNG-C electrode as the auxiliary electrode and a modified silver rod as the pseudo-reference electrode. To prepare the pseudo-reference electrode a silver rod was polished and dipped in a 0.1 mg/mL AgCl solution for 48 h. Afterwards, several polymeric layers were drop-casted on the rod tip. Firstly, three layers of 0.5 µL of a solution containing 74.8 mg/mL of polyvinyl butyral and 50 mg/mL of NaCl in methanol were drop-casted. Then, 0.3 µL of a 30 mg/mL polyurethane solution in tetrahydrofuran was added. Finally, the homemade pseudo-reference electrode, named as Ag-PVB-PU, was stored in 3 M KCl solution for 24 h, prior their use [46].

The continuous determination was carried out using a peristaltic pump working at 0.4 mL/min flow. A working potential of 0.02 V (vs. Ag-PVB-PU) was used, whereas lactate stock solutions with concentrations ranging from 0.2 to 20 mM (in 0.1 M PBS adjusted at pH 7.4) were used to calibrate the biosensor. Finally, synthetic samples were prepared with several salts and biocomponents to simulate an artificial interstitial fluid as follows: 0.1% albumin bovine serum, 4.7 mM glucose, 2.5 mM urea, 105 mM sodium chloride, 4 mM potassium chloride, 1.2 mM calcium chloride, 26 mM sodium bicarbonate and 1.7 mM sodium phosphate. These concentrations were selected to mimic the typical composition of human interstitial fluid. An adequate amount of 100 mM lactate stock solution was added to the samples to reach 2 and 4 mM of lactate. A calibration validation product for gas analyzer called GEM^®^ CVP 2 (1.75 mM LA) provided by Instrumental Laboratory Company (Bedford, USA) was used as a reference sample. This product was analyzed without dilution or any other treatment.

Human serum real samples were collected by specialized staff from different healthy adult volunteers in the University Hospital of Puerto Real (Cádiz, Spain). The serum was obtained by the centrifugation of the extracted blood at 3500 rpm for 5 min using serum separator tubes (SST) composed by a separating gel, based mainly on silicone, polyester and synthetic oils. In situ blood samples analysis were carried out with the gas analyzer.

## Results and discussion

### Biosensor manufacturing and stability studies of the redox mediator

To facilitate its suitability for human serum sample analysis, the proposed biosensor device incorporates a layer-by-layer configuration specifically intended to expand its linear operational range. The role of the polymeric layers is to control the flow of the lactate to the enzymatic layer to avoid the saturation of the enzyme and, consequently, enlarge the working linear range. Linear response at concentration values higher than 15 mM is desirable for applying this biosensor in blood samples, even in extreme situation when the theoretical maximum concentrations are reached [9].

The sensing principle of this biosensor is the oxidation of the L-lactate by the LOx and the formation of hydrogen peroxide (H_2_O_2_) as subproduct with the subsequent catalytic reduction of H_2_O_2_ by the PB (Figure S5), and the reduction at low potential of the oxidized organometallic complex. The oxidized phase of the PB, commonly known as Berlin Green, acts as a strong oxidizing agent and can be reduced by applying a low potential. Hence, the cascade reaction can be monitored using the chronoamperometry technique to observe the changes in the current intensity at a potential near 0 V. In addition, the use of this working potential diminishes interference issues [47].

First, Sonogel-Carbon electrodes were electrochemically activated in acid media and a layer of redox mediator Prussian Blue were drop-casted in their surfaces as described in Sect. 2.5, named them as Sonogel-Carbon-Prussian Blue (SNG-C-PB). A visual change in the electrode surface can be noticed. The stability of this layer in the working buffer solution was studied due to the instability of this metallic complex in neutral and basic media. The performances of two different configurations with polymeric layer drop-casted over the surface were studied as well to evaluate their capacity to protect the redox mediator. One of them has only a layer of Nafion, whereas another one has layers of chitosan, PVC and Nafion. The use of Nafion films in biosensor devices is a widely extended approach due to their features, such as biocompatibility or stability. However, the main advantage of the use of this polymer in biosensor manufacturing is their structural characteristic: a hydrophobic matrix with hydrophilic channel can be prepared. This membrane is selective, avoiding the flow of anionic interferents and hydroxyl groups to the inner part of the device that could degrade the PB layer. The configuration with only Nafion was tested due to the large number of references found in the literature using this approach [48].

Hence, electrochemical response of these configurations before and after 250 cyclic voltammograms in pH 7.4 buffer were compared. Equation 2 shows the electrochemical reaction that the redox mediator undergoes.

2 $$\mathrm{Fe}^2+\lbrack\mathrm{Fe}^2+{(\mathrm{CN})}_6\rbrack\leftrightarrow\mathrm{Fe}^3+\lbrack\mathrm{Fe}^2+{(\mathrm{CN})}_6\rbrack+\mathrm e^-$$

**Fig. 1:**
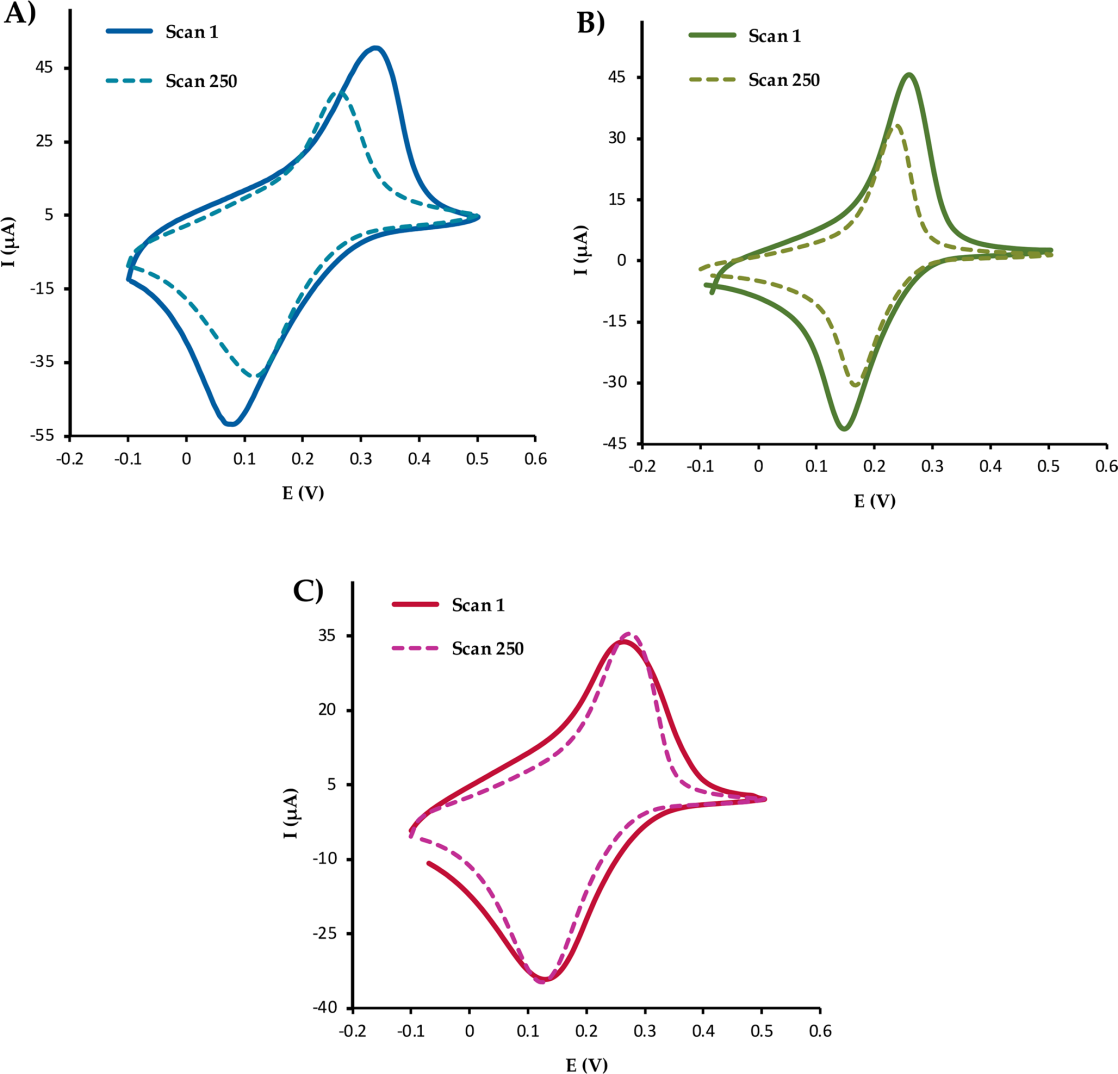
Cyclic voltammograms in PBS 0.1 M and pH 7.4 at 50 mV/s recorded with SNG-C-PB electrodes **A**, SNG-C-PB electrodes with a Nafion layer **B**, and SNG-C-PB electrodes with chitosan, PVC and Nafion layers **C**

Two well-defined peaks corresponding to the oxidation and reduction process of the PB are obtained with the sensor configuration studied, as observed in Fig. 1. The SNG-C-PB configuration (Fig. 1A) suffered anodic and cathodic peaks shifts of 71 and 48 mV, respectively, after 250 cycles. Besides, the current peak intensity falls 25% for both peaks. This decline in the electroactivity of the sensor device suggests a change in the PB layer, such as degradation or leaching of part of the material. A modification of the electrode with a coating layer of Nafion moderates the peak shifts to 20 mV, although the current peak intensity reduction remains at 27%. Finally, a configuration with layers of chitosan, PVC and Nafion was tested. In contrast, peak shifts of only 10 mV and current intensity fall of 5% for anodic peak and 2% for cathodic peak were observed in this case. These results suggest that the polymeric layer of Nafion avoids degradation or the phase converse of PB, but fails to retain the electrochemical activity of the platform. In contrast, the multiple layers configuration operates correctly after an extended time of application. Therefore, the arrangement of multiple polymeric layers was selected as the optimal configuration for further studies.

Next, a mixed solution of gold sononanoparticles (AuSNPs) and lactate oxidase (LOx) was drop-casted onto the PB layer to act as the sensing recognition element. The combination of these units has been proposed previously as a suitable approach to improve the LOx enzyme performance. Briefly, AuSNPs promote electron transfer and improve the catalytic activity of the biological recognition agent [33, 48]. These sononanoparticles stand out thanks to their fast and eco-friendly synthesis method assisted by high-energy ultrasound irradiation as well as by their small average particle size. The AuSNPs prepared for this application were characterized using dynamic light scattering and UV-Vis spectrophotometry. The corresponding results are shown in Figure S6. Briefly, the colloidal solution had a normal distribution with sizes that range from 2 to 18 nm and an average size of 6 ± 1 nm, whereas an absorption peak, corresponding to the AuNPs typical surface plasmon resonance band, was found at 520 nm in the UV-Vis spectra, as expected [49].

Finally, the AuSNPs-LOx layer on the electrode surface was covered by drop-casting the polymers mentioned beforehand: chitosan, PVC and Nafion. The surface of the prepared biosensor, named SNG-C-PB-LOx-AuSNPs-Chit-PVC-Nafion. Interestingly, the pink-purple color can be attributed to the AuNPs, whereas the polymeric layers can be spotted as well. The chitosan layer prevents enzyme leaching by entrapping it. Moreover, this biocompatible polymer confers additional resistance to the sensing composition [50]. The large number of intrinsic nitrogen- and oxygen-based functional groups included in the chemical structure of chitosan serve as starting points for covalent bonds. This feature led to the cross-linking of the chitosan film with other polymeric layers, giving as a result a consolidated multi-layer composite. The enzymes could be entrapped and protected with this intricate bio-network as well [51]. Further, the PVC acts as a diffusion membrane to limit the arrival of the lactate from the working solution to the enzyme, as well as to ease the oxygen flow [52]. Nafion avoids the diffusion of anionic interferents by producing a structure with hydrophilic channels within a hydrophobic matrix that acts as a selective barrier [53].

Electrochemical impedance spectroscopy was performed in order to evaluate how the sequential deposition of different layers influences the conductivity of the electrochemical biosensor. Figure S7 shows the Nyquist plots obtained for the SNG-C electrodes after modifications with different components used in the fabrication of the lactate biosensor. The spectrum obtained shows two characteristic regions: at high frequencies, a semicircle is observed, which reflects the kinetics of electron transfer at the electrode surface. At lower frequencies, a straight line appears, which is related to the diffusion of electroactive species in the electrolyte. The progressive changes observed in these regions provide useful information about how each modification step alters the interfacial properties of the electrode thereby affecting the sensitivity and overall behavior of the biosensor [54–56]. The impedance data obtained are showed in Table 1; Rs corresponds to the resistance of the electrolyte, Rp represents the resistance to charge transfer, and CPE accounts for the double-layer capacitance at the electrode/electrolyte interface.

**Table 1:** Electrical parameters calculated from the Nyquist plots in 0.5 mol L^− 1^ KCl solution containing 5 mmol L^− 1^ of Fe(CN)_3_^3-/4-^ for SNG-C, SNG-C-PB, SNG-C-PB-AuNPsLOx, SNG-C-PB-AuNPsLOx-Chit, SNG-C-PB-AuNPsLOx-Chit-PVC and SNG-C-PB-AuNPsLOx-Chit-PVC-Naf electrodes

Lactate biosensor composition	Electrolyte resistance (Rp) (Ω)	Solution resistance (Rs) (Ω)	CPE (µF)
SNG-C	208.3	272.3	6.34
SNG-C-PB	72.8	440.7	30.3
SNG-C-PB-AuNPsLOx	179.7	775.7	3260
SNG-C-PB-AuNPsLOx-Chit	560.7	228	519.3
SNG-C-PB-AuNPsLOx-Chit-PVC	563	243	23.43
SNG-C-PB-AuNPsLOx-Chit-PVC-Naf	589.3	228.3	21.4

According to the results presented in Table 1, the incorporation of the Prussian Blue layer significantly enhances the conductivity of the biosensor. This improvement is reflected in the decrease in charge transfer resistance, a key parameter in electrochemical impedance. The enhanced performance might be attributed to the intrinsic properties of the redox mediator, mixed valance compound with highly open cubic lattice structure. In this network, iron (II) and (III) are linked through by bridges (-CN-), generating a rigid three-dimensional framework that contains interstitial cavities. These can accommodate water molecules and small cations like K^+^, which can move in and out of the lattice during redox process. The reversible transition between Fe (II) and (III) coupled to this ion insertion/extraction mechanism facilitates rapid ion transport, while the Fe-CN-Fe backbone provides an efficient way for electronic conduction [57–59]. Nevertheless, deposition of polymeric layers may lead to an increase in the electrode charge transfer resistance. This is due to these layers acting as a physical barrier, limiting the access of the redox species to the conductive surface [60]. In addition, polymeric coatings can also restrict ionic transport, hindering efficient interaction between the electrode and the electrochemical medium. As result, an increase in the Rp value is observed when polymer layers are added to the biosensor. This effect is particularly important in biosensor design, as polymer layer can provide selectivity and stability, but simultaneously and overall electrochemical response of the device. Furthermore, a more in-depth analysis was performed using the Z-view software to calculate the fitted values of Z’ and Z’’, allowing the determination of chi-squared (χ²) values. In all cases, χ² values were below 0.05, indicating that there were no significant differences between the experimentally obtained values and those predicted by Z-view using the equivalent circuit shown in Figure S7 (see Supplementary Material).

**Fig. 2:**
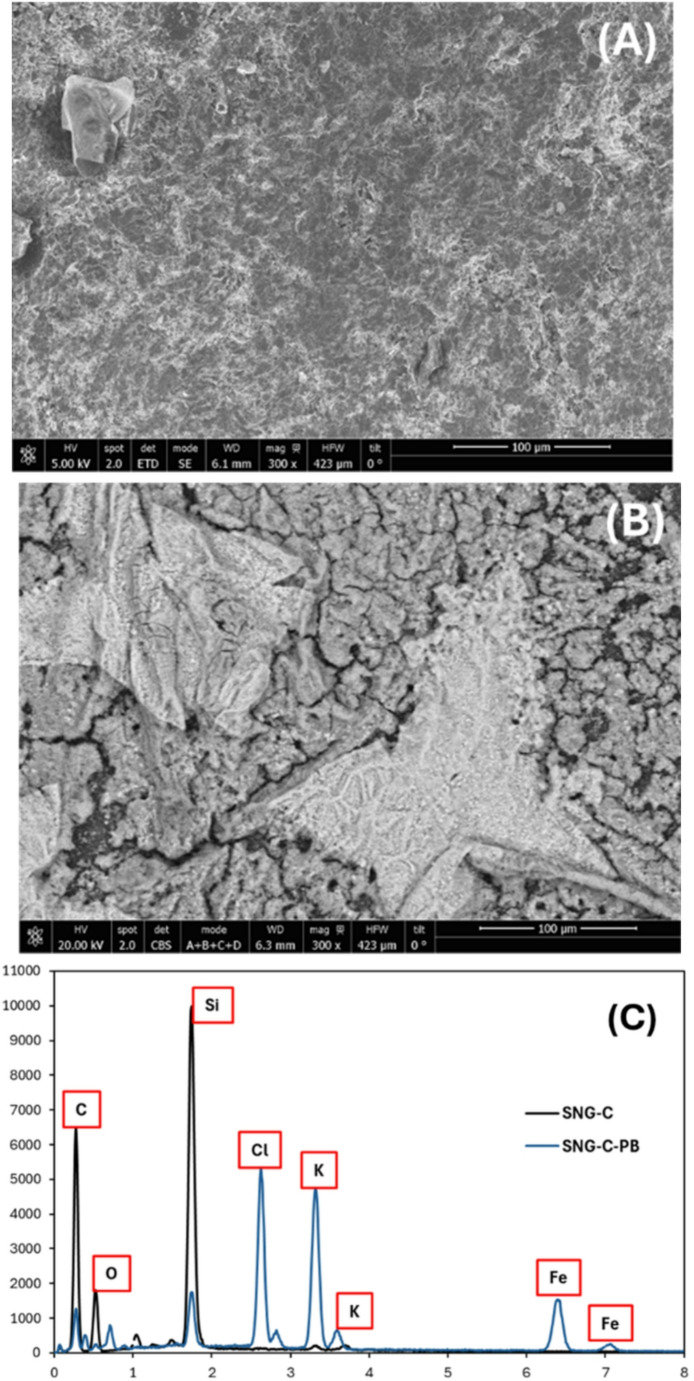
SEM micrographs of: **A** SNG-C (secondary electron detector), **B** SNG-C-PB (backscattered electron detector) and **C** EDS spectrum of the SNG-C (black) and SNG-C-PB (blue) electrodes

**Fig. 3:**
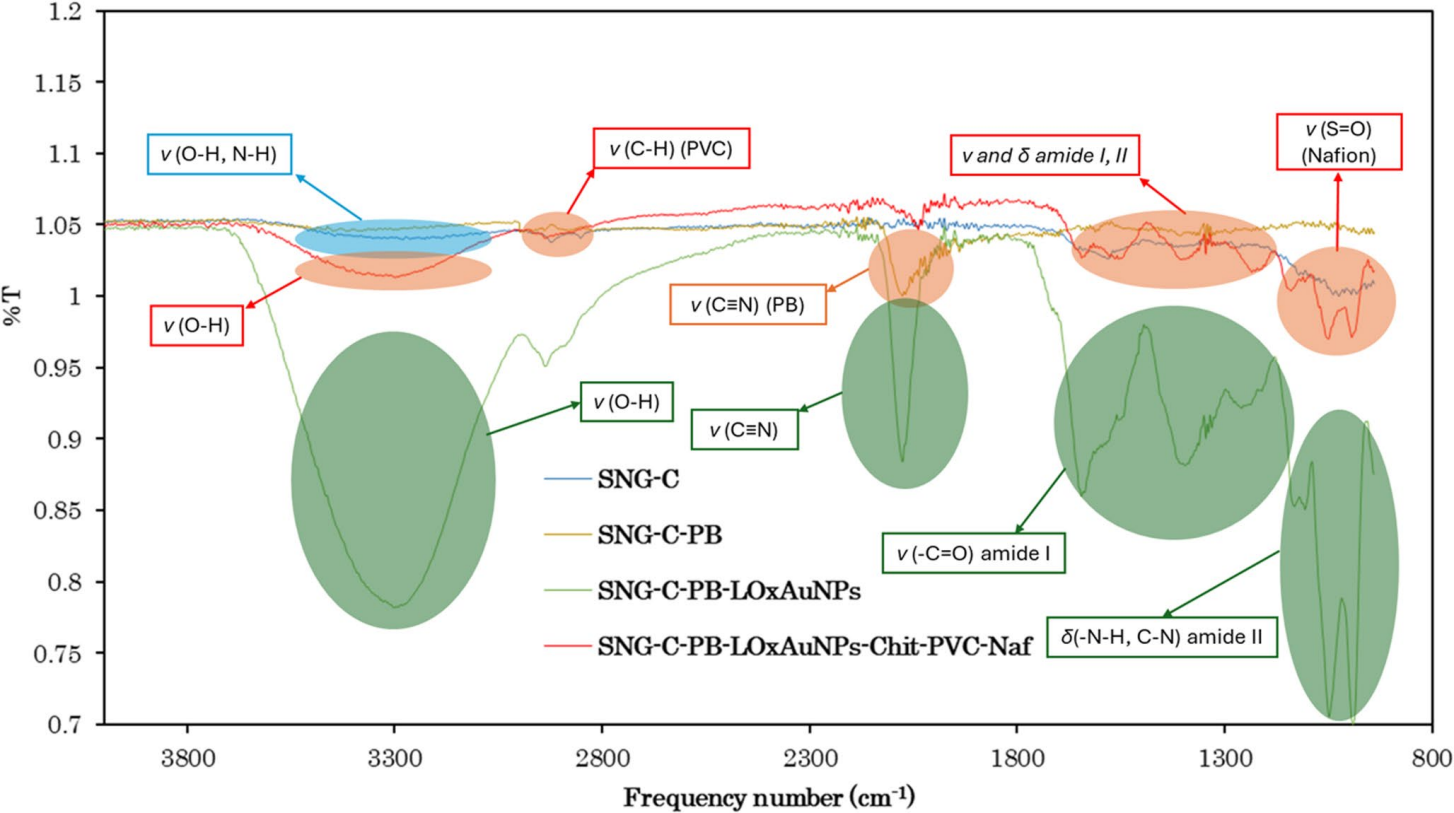
FTIR spectra of the modified electrodes: SNG-C (dark blue), SNG-C-PB (orange), SNG-C-PB-AuNPsLOx (green), and SNG-C-PB-AuNPsLOx-Chit-PVC-Naf (red). The main absorption bands are assigned as follows: the S = O stretching vibration of Nafion at approximately 1050 cm⁻¹, the C–H stretching bands of PVC near 2900 cm⁻¹, the O–H and N–H stretching vibrations of chitosan around 3400 cm⁻¹, the amide I and II bands of the enzyme LOx at ~ 1650 cm⁻¹ and ~ 1550 cm⁻¹, respectively, and the C ≡ N stretching vibration of Prussian Blue at ~ 2070–2100 cm⁻¹. The spectra were recorded with a resolution of 2 cm⁻¹

In Fig. 2, the SEM image of the unmodified SNG-C electrode surface shows a rough and heterogeneous morphology with noticeable carbon agglomerates of different sizes and shapes, creating an irregular and porous texture. This topography is characteristic of the sonogel matrix and provides natural pathways for electron transfer [61]. In contrast, the SEM image of the SNG-C-PB electrode reveals a clear modification of the surface morphology, where crystalline structures typical of Prussian Blue (PB) can be observed [62]. We should take into account that the backscattered electron detector has been used in this case to highlight the contrast of high atomic weight elements (like Fe, light grey; darker zones correspond to the Sonogel-Carbon transducer); hence, as observed, the whole surface is covered by a PB film. These PB structures, usually granular or cubic in form, adhere to and almost fully cover the carbon matrix, giving rise to a more complex and textured surface. Complementary evidence is provided by the EDS analysis: the spectrum of the unmodified SNG-C electrode shows peaks corresponding mainly to silicon (Si) and oxygen (O), which can be attributed to the Si-O matrix, and C to the methyl groups of the silane precursor and the graphite employed as massive modifier of the SNG-C material. However, the spectrum of the SNG-C-PB electrode displays two additional peaks associated with iron (Fe), confirming the presence of Prussian Blue, since iron is a key element in its structure. Furthermore, peaks corresponding to potassium (K) and chlorine (Cl) are also detected, which can be explained by the usage of K₄[Fe(CN)₆], K₃[Fe(CN)₆], KCl and HCl during the synthesis, leaving residual ions on the electrode surface. Taken together, the SEM images and EDS spectra confirm the successful immobilization of Prussian Blue on the electrode surface and also demonstrate an effective integration between the modifier and the carbon substrate, thereby enhancing the electrode potential for electrochemical applications. Following the electrochemical study, together with the analyses carried out by Scanning Electronic Microscopy (SEM) and energy-dispersive X-ray spectroscopy (EDS), additional characterization was undertaken using atomic force microscopy (AFM) and Fourier-transform infrared spectroscopy (FTIR) in order to better understand the structure of the polymeric layers in the biosensor (Fig. 3). AFM analysis (Figure S8) of the SNG-C-PB-Lox-AuNPs-Chit-PVC-Nafion biosensor revealed surface roughness parameters of R_a_ = 2.454, S_sk_ = 0.8209, and S_ku_ = 2.607. The relatively low R_a_ value indicates a smooth and compact surface, consistent with the presence of polymeric layers that act as a barrier to lactate diffusion and help prevent enzyme saturation. Meanwhile, the positive S_sk_ and S_ku_ values suggest that, although the surface is overall leveled, minor peaks and valleys are still present. As complementary surface characterization, Fourier transform infrared spectroscopy (FTIR) was performed to confirm the presence and interactions of the different components within the multilayered biosensor (Fig. 3). The spectra displayed characteristic absorption bands of chitosan (O-H and N-H stretching around 3400 cm^−1^, and amide I and II bands at ~ 1650 and 1550 cm^−1^), Nafion (S = O stretching at ~ 1050 cm^−1^), and PVC (C-H, stretching near ~ 2900 cm^−1^), together with additional contributions from the enzyme.

In particular, the amide I and II bands confirm the presence of lactate oxidase, while the slight variations in their intensity and position indicate its interactions with AuNPs, consistent with enzyme immobilization on the metal nanostructures.

The FTIR spectra also revealed the characteristic PB band at ~ 2070–2100 cm⁻¹, corresponding to the C ≡ N stretching vibration of the Fe–CN–Fe framework, together with signals around 1600 cm⁻¹ and 3400 cm⁻¹ related to interstitial water molecules within the PB lattice.

Additionally, in the case of the Sonogel–Carbon support, a weak band was observed near 3400 cm⁻¹, which can be attributed to hydroxyl groups generated during the electrode activation process, as described in Sect. 2.3. Altogether, these findings corroborate the successful integration of the polymeric matrix, PB mediator, AuNPs, and the enzyme into a single hybrid structure, confirming both the chemical composition and the functional interactions necessary for efficient biosensor performance [42, 63–71].

An adequate performance of the prepared biosensor in a wide linear range that covers the LA concentrations found in human serum (0.5–2 mM in normal conditions, up to 20 mM in extreme exercise or unhealthy conditions) is desired. The accomplishment of this objective was validated through the calibration of lactate using the developed biosensor. It is remarkable as well as the price of each fabricated biosensor. Each bare SNG-C electrode has an estimated cost of 0.35 €, whereas all the reagents used in the layer arrangement have negligible prices, except the enzyme. Considering an approximate price of 240 € per LOx enzymes bottle (100 U), each enzymatic layer (2.12 U) costs 5.08 €. Hence, the total cost of each biosensor device is approximately 5.44 €.

### Calibration of lactate with the biosensor developed

**Fig. 4:**
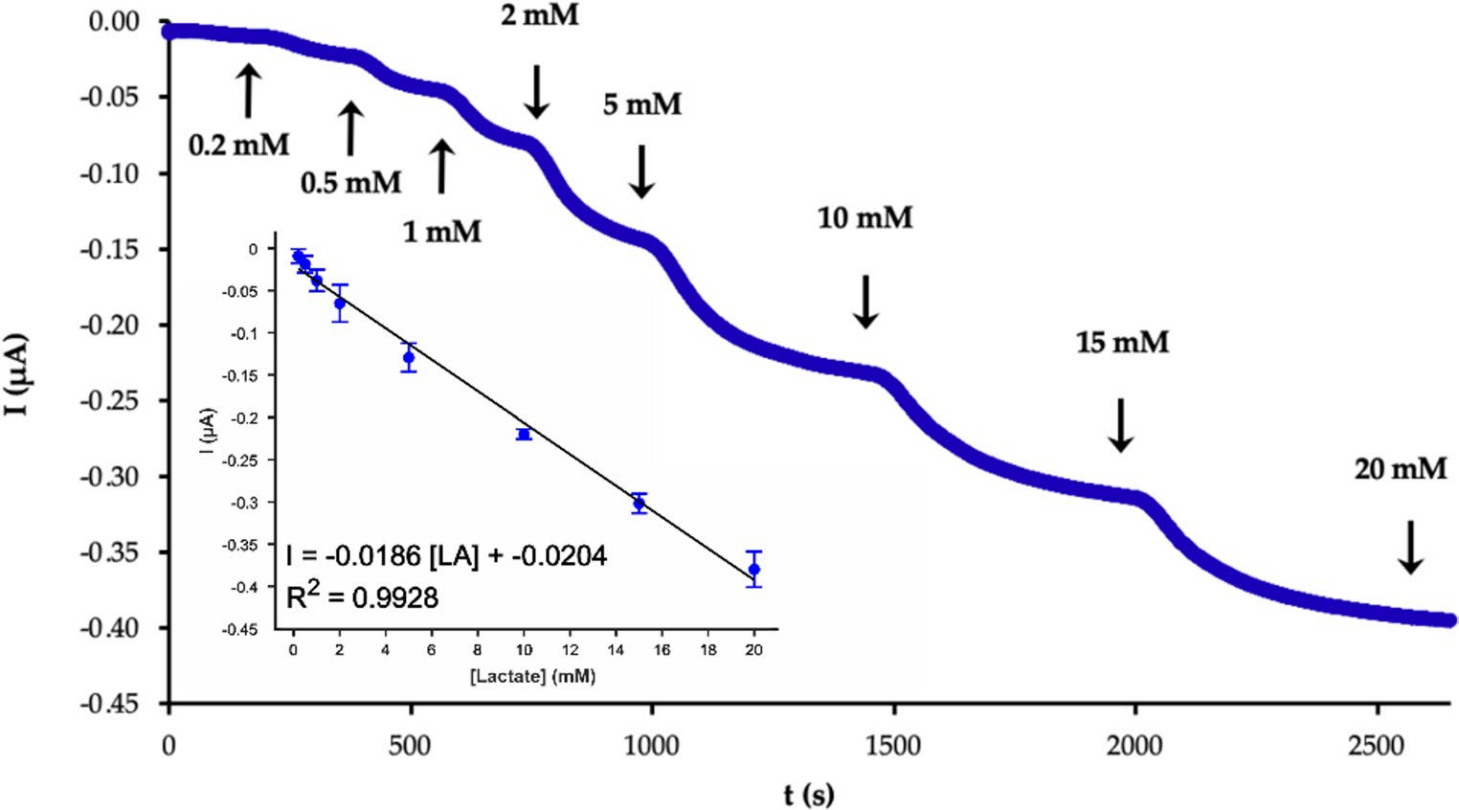
Chronoamperograms corresponding to the calibration of LA with the SNG-C-PB-LOx-AuSNPs-Chit-PVC-Nafion biosensor device in batch regime. The inset displays the calibration plot obtained. The buffer solution was 0.1 M PBS pH 7.4 and the working potential was 0.10 V (vs. Ag/AgCl)

Analytical calibration of lactate with the developed biosensor in batch mode was carried out using the chronoamperometry technique at a working potential of 0.1 V (vs. Ag/AgCl). The hydrogen peroxide produced by the oxidation of lactate by the enzyme leads to an oxidation of the PB layer. The reduction of the redox mediator at this working potential gives place to an increase of the cathodic current. Hence, the current of the chronoamperometric signal decrease with each addition of lactate, as shown in Fig. 4. An excellent relationship (R2 = 0.9928) between current intensity and LA concentration can be established in the range of 0.2–20 mM. The linear regression equation (Eq. 3) in this case was:

3 $$\begin{array}{l}I\left(\mu A\right)=-\left(0.0186\pm0.001\right)\left[LA\right]\\\left(mM\right)-\left(0.0204\pm0.015\right)\end{array}$$

The sensitivity value provided by the SNG-C-based biosensor was 1.74 ± 0.12 µA mM^−1^ cm^−2^ (*n* = 3). The limit of detection of this sensor device, calculated as three times the standard deviation of the blank divided by the slope (LOD = 3 sq/m), was 67 ± 7 µM. Therefore, this biosensor device is suitable to be applied in the analysis of LA in blood samples, with concentrations ranging from 0.5 to 15 mM or even 20 mM in extreme conditions [72]. The wide working linear range can be attributed to the polymeric layers acting as a diffusion limiting membrane.

Furthermore, the figures of merits of this SNG-C-based biosensor were compared to the electroanalytical performance of other biosensor devices previously reported in the literature. Research works of the last years have been chosen for this comparative discussion as shown in Table 2, and all of these devices use the combination of LOx and PB as recognition element. These lactate biosensors can be classified by their working linear ranges. On the one hand, those configurations that use LOx without substantial protection membrane show high sensitivities values at the expense of maximum working concentrations lower than 5 mM. On the other hand, the biosensor configuration that includes the use of membrane diffusion, comprises of polymers such as chitosan, exhibited linear relationship at higher LA concentrations. The main drawback in these cases is the lower sensitivity values obtained compared to the biosensor applied at lower concentration ranges. In this sense, our SNG-C modified device exhibits a sensitivity higher than most of the devices reported or comparable to the rest, such as the screen-printed modified biosensor reported by Zanardi et al. Our LOD is also lower than most cases and adequate for the application in blood and serum samples analysis (lower LA levels found in these kinds of samples are around 0.5 mM [73]). Finally, although wider linear ranges are found in the literature up to 50 mM, our working range is competent for the desired application in the monitoring of LA in patients.

**Table 2:** Comparison of the analytical performance of biosensors based on lox and PB in the determination of LA

Sensor	Working Potential (V)	Linear range (mM)	Sensitivity (µA mM^−1^ cm^−2^)	LOD (µM)	Time response (s)	Ref
LOXENs/PB/SPCE	−0.10	0.001–0.01	15.9	3.1	5	[73]
LOx/PB nanocubes	−0.05	0.01–0.5	6.38	10	N.A.	[74]
e-LOx/PB/C-SPE	−0.10	0.1–1	N.A.	70	4	[75]
CP-PB-AvLOx S175A@Ch	0.00	0.2–2	17.52	38	50	[76]
SPCE-PB/Fe_3_O_4_@PDA-LOx	−0.15	0.1–4.62	1.54	320	120	[77]
SPE/Graphene/PB/PVA-SbQ-LOx	−0.10	0.25–5	1.6	-	N.A.	[78]
SPCE-PB-LOxAuNPs-Chit-PVC-Naf	0.05	0.5–20	0.13	180	20	[26]
SPCE-PB/Nafion/LOx	−0.20	1–24	N.A.	1310	N.A.	[79]
SPCE/PB/rGO/Au/LOx/Chit	−0.05	0.222–25	1.9	0.8	20	[47]
CE-fSWCNTs/Chit-PBNPs/Chit LOx	−0.17	1–25	N.A.	200	40	[63]
Au-PB-LOx-Chit/CNts	-	0–30	3.10	-	10	[64]
PB-LOx-GO-Chit-SPCE	−0.05	1–50	0.39	0.028	60	[65]
SPCE-PB-Lox/BSA/Nafion-ETH500/PVC/DOS	−0.05	1–50	N.A.	110	30	[66]
SNG-C-PB-LOx-AuSNPs-Chit-PVC-Nafion	0.10	0.2–20	1.74	67	19	This work

*LOD* Limit of detection, *LOx* Lactate oxidase, *PB* Prussian Blue, *LOXENs* Mixture of LOx and reduced graphene oxide, *SPCE* Screen printed carbon electrode, *Cu*-*MOF* Copper metallic framework, *CS* Chitosan, *e*-*LOx* electrospray deposited LOx, *CP* Carbon paper, *AvLOx* *S175A* LOx from *Aerococcus viridans,* S175A mutant, *Ch* Chitosan, Fe_3_O_4_@PDA: Magnetite nanoparticles coated with polydopamine, *SPE* Screen printed electrode, *PVA*-*SbQ* Poly(vinyl alcohol) bearing styrylpyridinium groups, *AuNPs* Gold nanoparticles, *PVC* Polyvinyl chloride, *Naf* Nafion, *rGO* Reduced graphene oxide, *Chit* Chitosan, *CE* Carbon electrode, *fSWCNTs* functionalized single-walled carbon nanotubes, *PBNPs* Prussian Blue nanoparticles, *CNts* Carbon nanotubes, *GO* Graphene oxide, *BSA* Bovine serum albumine, *ETH500* Tetradodecylammonium tetrakis(4-chlorophenyl)borate, *DOS* bis(2-ethylhexyl) sebacate, *AuSNPs* Gold sononanoparticles. *N*.*A*. Not applicable

### Michaelis-Menten kinetics study

**Fig. 5:**
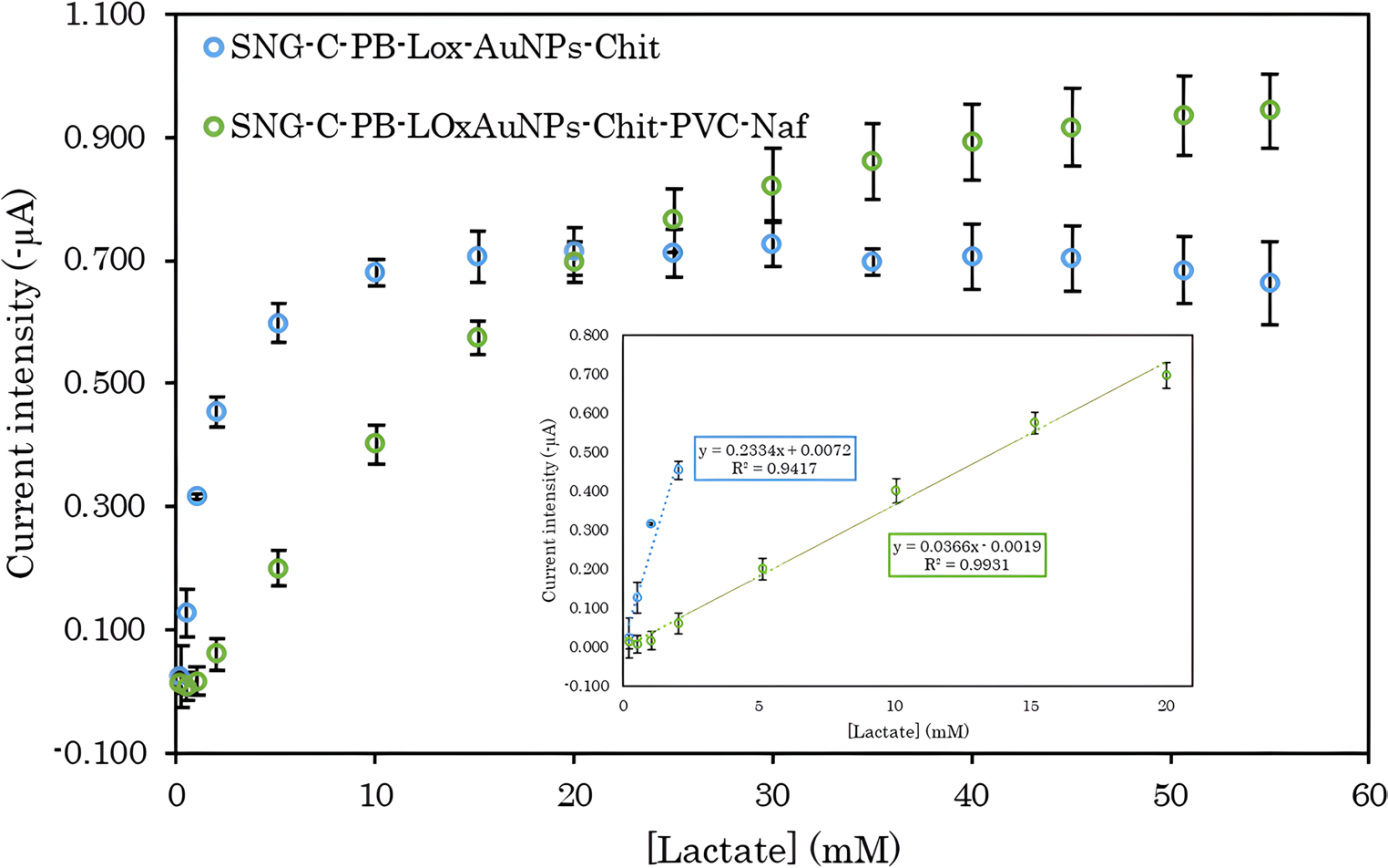
Michaelis–Menten kinetic analysis of the lactate biosensor in 0.1 M PBS. The amperometric response was measured over a lactate concentration range of 0.2 to 55 mM. A linear response was observed from 0.2 to 2 mM for the chitosan-only biosensor and from 0.2 to 20 mM for the complete biosensor with polymeric layers. Measurements were performed in triplicate, and data points represent the mean ± standard deviation. Nonlinear regression fitting to the Michaelis–Menten equation was used to determine the apparent Michaelis constant (K_m_) and the maximum current (I_max_) for each biosensor configuration

The kinetics of the lactate biosensor with and without diffusion membrane were investigated following the classical Michaelis-Menten model, as it was described in Sect. 2.7 and widely reported in previous studies on enzymatic biosensors [80–84]. The amperometric response of the biosensor was evaluated against increasing lactate concentrations, starting from 0.2 mM until enzyme saturation was observed, both for the biosensor with the enzymatic layer supported by chitosan and for configurations incorporating diffusion multipolymeric layer, (Fig. 5). Linear range was also plotted for comparison purposes (Fig. 5 inset). The data were fitted to the Michaelis-Menten equation using nonlinear regression, allowing the determination of key cinetic parameters K_m_ and I_max_, which reflect the affinity of the immobilized enzyme for the substrate and the maximum response capacity of the system, respectively. Importantly, it is well known that I_max_ is proportional to kinetic maximum speed for amperometric biosensors. Concerning, biosensors containing only the redox mediator, the enzymatic mixture, and chitosan (SNG-C-PB-LOxAuNPs-Chit), the obtained values were K_m_ = 1.59 mM and I_max_ = 0.727 µA, whereas for the complete lactate biosensor with diffusion multipolymeric layer (SNG-C-PB-LOxAuNPs-Chit-PVC-Naf), the corresponding values were K_m_ = 12.93 mM and I_max_ = 0.943 µA. These results indicate that the presence of polymeric layers such as PVC and Nafion limits substrate access to the enzyme, resulting in a higher K_m_, which effectively prevents enzyme saturation and allows an extension of the linear working range of the biosensor. In contrast, the notably low K_m_ observed for the biosensor containing only chitosan can be attributed to the greater accessibility of lactate to the enzyme, as there are minimal polymeric layers restricting substrate diffusion, allowing the enzyme to reach saturation more readily at lower substrate concentrations. To further support the validity of these findings, the values of K_m_ and I_max_ were calculated using three different theoretical models (Table 3), such as Lineweaver-Burk [84, 85], Eadie-Hofstee [85] and Hanes-Wolf [86]. In all three approaches, the K_m_ values were consistently higher for the biosensor incorporating all polymeric layers than for the chitosan-only biosensor, thereby confirming our hypothesis. Remarkably, the results obtained from the three theoretical models were in good agreement with the experimental data: the K_m_ values for the chitosan-only biosensor remained lower than those of the complete biosensor, and the values predicted by the models were similar to those measured experimentally. These observations are also in agreement with previous reports in the literature [82, 83, 87, 88], providing additional support for our interpretation of the role of polymeric layers in modulating substrate accessibility and extending the linear working range of the biosensor. Notably, the saturation behavior of the biosensors reveals significant differences that complement the kinetic parameters. In the chitosan-only sensor, saturation begins to appear at substrate concentrations around 5 mM, indicating a relatively narrow linear working range. In contrast, the multilayer biosensor maintains a nearly linear response up to approximately 25 mM, demonstrating that the additional polymeric layers effectively delay saturation. This extended linear range is particularly relevant for practical applications, as it allows for accurate quantification over a wider concentration span. The combination of higher K_m_ and I_max_ values with the delayed onset of saturation confirms that not only does the polymeric architecture modulate enzyme kinetics, but it also enhances substrate accessibility and overall sensor performance [89–95].

**Table 3:** Comparative analysis of K_m_ and I_max_ kinetic parameters obtained from triplicate experimental measurements in lactate biosensors SNG-C-PB-LOxAuNPs-Chit and SNG-C-PB-LOxAuNPs-Chit-PVC-Naf, and their contrast with the values predicted by three different theoretical models

Biosensor	Model	K_m_ (mM)	I_max_ (µA)
SNG-C-PB-LOxAuNP-Chit	Experimental (*n* = 3)	12.93 ± 0.97	0.727 ± 0.06
	Lineweaver-Burk	21.50	0.739
	Eadie–Hofstee	16.6	1.33
	Hanes-Wolf	29.35	0.719
SNG-C-PB-LOxAuNP-Chit-PVC-Naf	Experimental (*n* = 3)	1.59 ± 0.095	0.943 ± 0.04
	Lineweaver-Burk	1.36	0.313
	Eadie–Hofstee	0.74	1.25
	Hanes-Wolf	1.57	1.30

### Establishing figures of merits of the developed biosensor

Several analytical parameters of the SNG-C-based biosensor were assessed to evaluate the quality of this device. Firstly, a repeatability value of 4.73% was obtained by performing three different calibrations using the same biosensor and calculating the RSD% of the sensitivities obtained. Additionally, the RSD% of the sensitivity values of three different biosensors was used to calculate a reproducibility of 3.98%. These results evidence the durability of the biosensors after the calibrations and their reusability, as well as the robustness of the fabrication methodology. Another crucial parameter is the lifetime of the developed device. Electroanalytical response was recorded in 2 mM LA solution several days using the same biosensor to get information about the impact of storage in their performance. The electrodes were generally stored in 0.1 M PBS solution at pH 7.4, in darkness and at 4 °C. The response was constant until the 15th day of the study, but successive measures suffered a fall in the current intensity. This decrease could be ascribed to the degradation of the LOx enzymes and a loss of their enzymatic activity. Therefore, the mean lifetime of the SNG-C-PB-LOx-AuSNPs-Chit-PVC-Nafion is 13 days (Figure S9). It is noteworthy that the polymeric layers drop-casted onto the sensing element protect it and prevent the leaching of the LOx enzymes, enlarging the functioning period of the biosensor device. As mentioned in Sect. 2.7, the mean lifetime of the biosensor was also evaluated in real human serum samples to enhance clinical translation. Each day, one serum sample (analyzed in triplicate) with a lactate concentration determined by the hospital reference method was measured. The obtained results indicate that the proposed biosensor is capable of accurately detecting lactate in human serum for at least 6 days, with a recovery factor around 98% during this initial period. However, by the seventh day the recovery factor dropped to approximately 40%, confirming the malfunctioning of the biosensor. Hence, the biosensor stability in serum is lower than that observed in PBS for detecting a theoretical concentration of 2 mM. This reduced stability is likely due to the more complex matrix of human serum and the varying lactate concentrations from day to day (normal and after effort conditions), which may account for the shorter operational lifetime in real samples.

Next, the stability of the measure was assessed by recording the long-term response of the biosensor in a buffer solution. Any substantial increase, decrease or drift was observed in the recorded current intensity after measuring it for 8 h, as observed in Figure S10. The stability shown by the biosensor device makes it suitable for continuous monitoring of lactate over long periods. In the case of 1 mM lactate, the signal variation was − 1.81 × 10⁻² µA in artificial serum and − 1.40 × 10⁻² µA in PBS. At the higher concentration of 15 mM, a greater variation was observed due to the more extreme working conditions, with current intensity changes of − 8.5 × 10⁻² µA in PBS and − 1.27 × 10⁻¹ µA in artificial serum. These results clearly justify the high stability of the sensor performance under both normal and extreme conditions in different matrices.

**Fig. 6:**
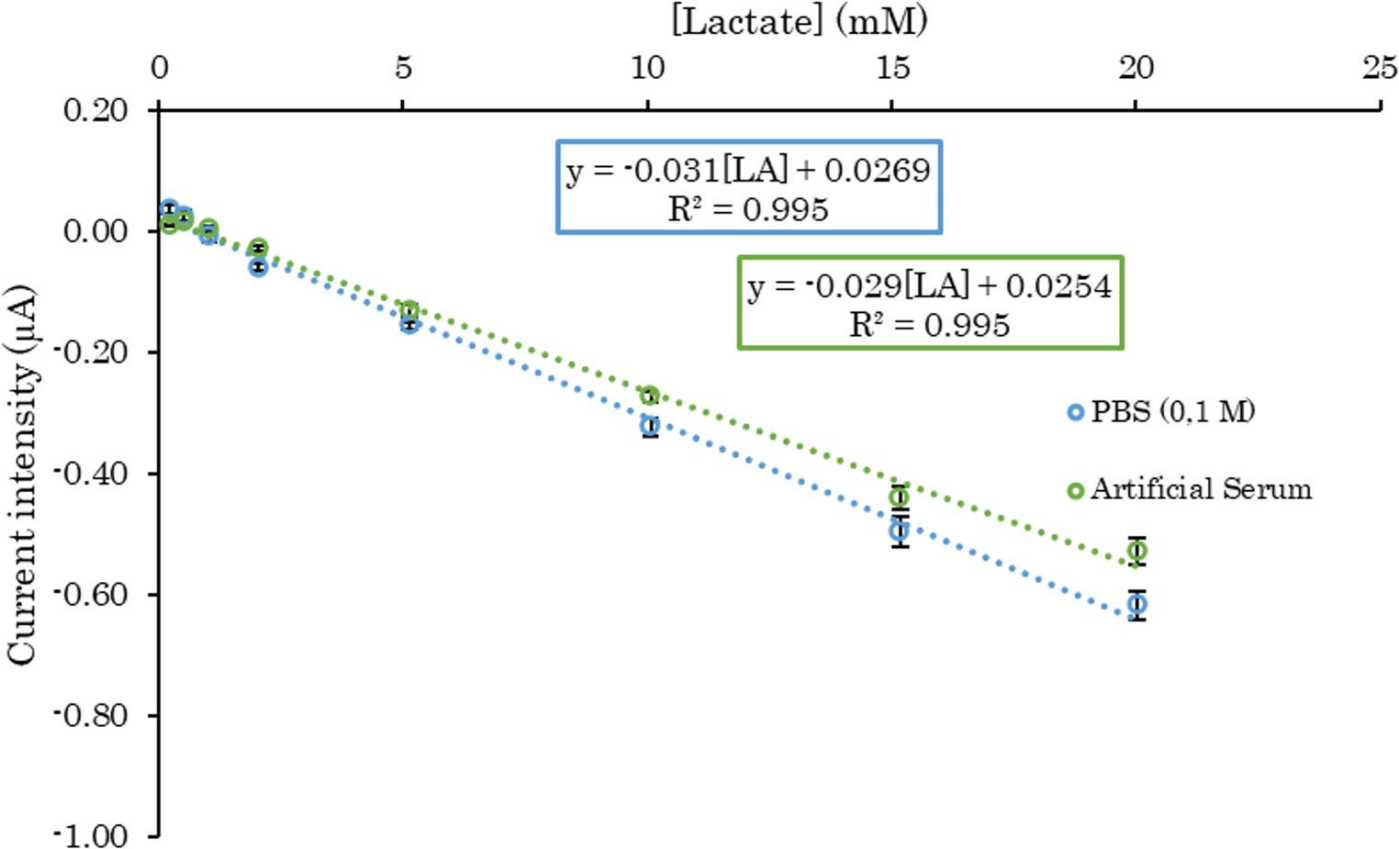
Calibration plots of LA with the SNG-C-PB-LOx-AuSNPs-Chit-PVC-Nafion biosensor device in absence (blue) and in presence (green) of several interferents (264 µM paracetamol, 4.33 mM bilirubin, 41 µM salicylate, 0.05 mg/mL hemoglobin, 100 µM ascorbic acid, 100 µM uric acid, 6 µM dopamine, 5 mM glucose, 20 mg/mL BSA, 3.5 mM KCl, 140 mM NaCl and 1.5 mM CaCl_2_. Data are presented as mean ± SD (*n* = 3 devices, 2 print batches). Error bars represent standard deviation

Finally, the biosensor selectivity was studied using several inorganic and organic compounds that can be found in physiological samples. Moreover, the concentrations of these interferents used are similar to those expected in these kinds of samples to simulate the conditions and test the applicability of the electrodes. These specifications can be found in Sect. 2.7. A 0.1 M PBS (pH 7.4) solution containing the mentioned interferents was prepared and the calibration of LA with the SNG-C-PB-LOx-AuSNPs-Chit-PVC-Nafion device was performed in this medium. The sensitivity obtained in this case was 4.7% lower than the one obtained by performing the calibration in normal PBS. Furthermore, the differences in slopes, y-intercepts, and current intensities at 2 mM lactate (the normal blood lactate concentration) between PBS and artificial serum were below 5% in all cases, demonstrating an excellent linearity and confirming the reliability of the biosensor under conditions simulating physiological samples. This negligible variation suggests that the biosensor exhibits adequate selectivity towards LA and could be applied in similar conditions as the one studied, such as physiological samples of serum or blood (Fig. 6). This ability can be attributed to the exceptional natural selectivity of the LOx enzyme, as well as to the selective control of the polymeric membranes of Nafion and the rest of layers. The next step in this work to further assess the applicability of the biosensor in the monitoring of LA in physiological samples is the design of an affordable and suitable flow cell for the prepared electrodes.

### 3D microfluidic cell development

One of the objectives of this work is the application of the developed SNG-C-based biosensor in the determination of LA in flow regime for the subsequent analysis of physiological samples. The main challenge, in this case, is the absence of commercial flow cells for SNG-C electrodes due to their handmade and innovative nature. Hence, the design and development of a novel flow cell was carried out to fulfil our objectives. A 3D printer was used for the fabrication of this device due to the versatility to assemble different shapes and the low cost of the manufacturing process.

Firstly, a three-electrodes configuration was considered as the primary specification. The developed SNG-C-PB-LOx-AuSNPs-Chit-PVC-Nafion biosensor acts as the working electrode, whereas an unmodified SNG-C electrode acts as the auxiliary electrode. Commercial Ag/AgCl reference electrode was discarded due to the large size. A slim silver rod was properly modified to act as pseudo-reference electrode instead (Ag-PVB-PU). Therefore, two openings of 1.55 mm for the SNG-C electrodes and another opening of 1 mm for the silver rod were planned. The next specification was that the chamber of the cell comprises the lowest possible volume of working solution. A triangle arrangement of the electrodes, proposed to have the same distance between each electrode, was rejected due to the wide chamber required and the presence of dead volume. Another rejected proposition was to arrange the electrode in three different canals, which lead to the requirement of higher volume of samples and the different times of arrival of the working solution to the electrodes. Finally, a single flow channel and a linear arrangement of the electrode were chosen as the best configuration for the proposed cell. The electrodes are positioned parallel to the working solution’s flow, with the biosensor centrally located and equidistant from both the auxiliary and pseudo-reference electrodes. Moreover, the 2 mm inter-electrode distance ensures the simultaneous arrival of the sample at the electrodes. The flow channel possesses a diameter of 2 mm and a longitude of 30 mm. Hence, the volume of sample required to fill the chamber of the cell is only about 280 µL.

The microfluidic cell was designed as two different pieces. The upper part acts as a container for the electrodes and has a similar height to protect them and confer robustness. The lower part contains the microflow channel, three holes for the electrodes and two openings where hollow 3D-printed screws with polytetrafluoroethylene tubes inside are connected to pump the working solution to the cell chamber. The polymer polyethylene terephthalate glycol (PETG) was selected as the filament to print the cell due to its properties: high resistance, durability, flexibility, density, excellent layer adhesion, chemical resistance to both acidic and alkali compounds, recyclability and biocompatibility [68], with recent studies further confirming that PETG is non-cytotoxic and meets biocompatibility standards (ISO 10993-5:2009 and ISO 10933-12:2021), making it suitable for biomedical applications [96, 97] Printing specifications are explained in Sect. 2.7, whereas the sizes of the pieces are reported in Figures S1, S2, S3 (A and B) and S4 of the supplementary material.

The main highlights of this novel microfluidic cell are its simplicity, reduced working volume, affordability, eco-friendly nature and fast fabrication. 3D printing of the different part of this device takes around 8 h and can be performed in situ, without additional extended commercialization and transporting times. The total cost of the cell fabrication can be calculated using the printing time, filament mass used (around 41 g) and filament roll cost, printer output power (160 W) and electricity cost. Hence, the cost of each printed microfluidic cell is only around 1.5 €. This affordable cost is even more impressive if it is taken into account that the price of a commercial flow cell for screen-printed electrodes is around 700 €, three hundred times more expensive than our handmade device. Furthermore, the recyclability of the PETG filament use for the fabrication and the absence of other reagents for this process confer this device an eco-friendly nature. Next, the designed microfluidic cell was tested using the developed biosensor for analytical applications.

### Electrochemical monitoring of lactate in flow regime

The handmade pseudo-reference electrode was used to determine which working potential should be applied during the chronoamperometric measurements in the flow regime. Cyclic voltammograms were recorded in a [Fe(CN)_6_]^−3^ solution with the handmade pseudo-reference electrode (Ag-PVB-PU) and a commercial Ag/AgCl (3 M KCl) one (Figure S11) to observe the potential shift between both signals. Oxidation peak potentials of the hexacyanoferrate system were 0.21 V, when the handmade electrode was used, and 0.28 V when recording using the commercial reference electrode. A shift of 0.08 V between each case is calculated. Hence, a working potential of 0.02 V is applied during the measurements in flow regime instead of 0.1 V used in batch mode.

**Fig. 7:**
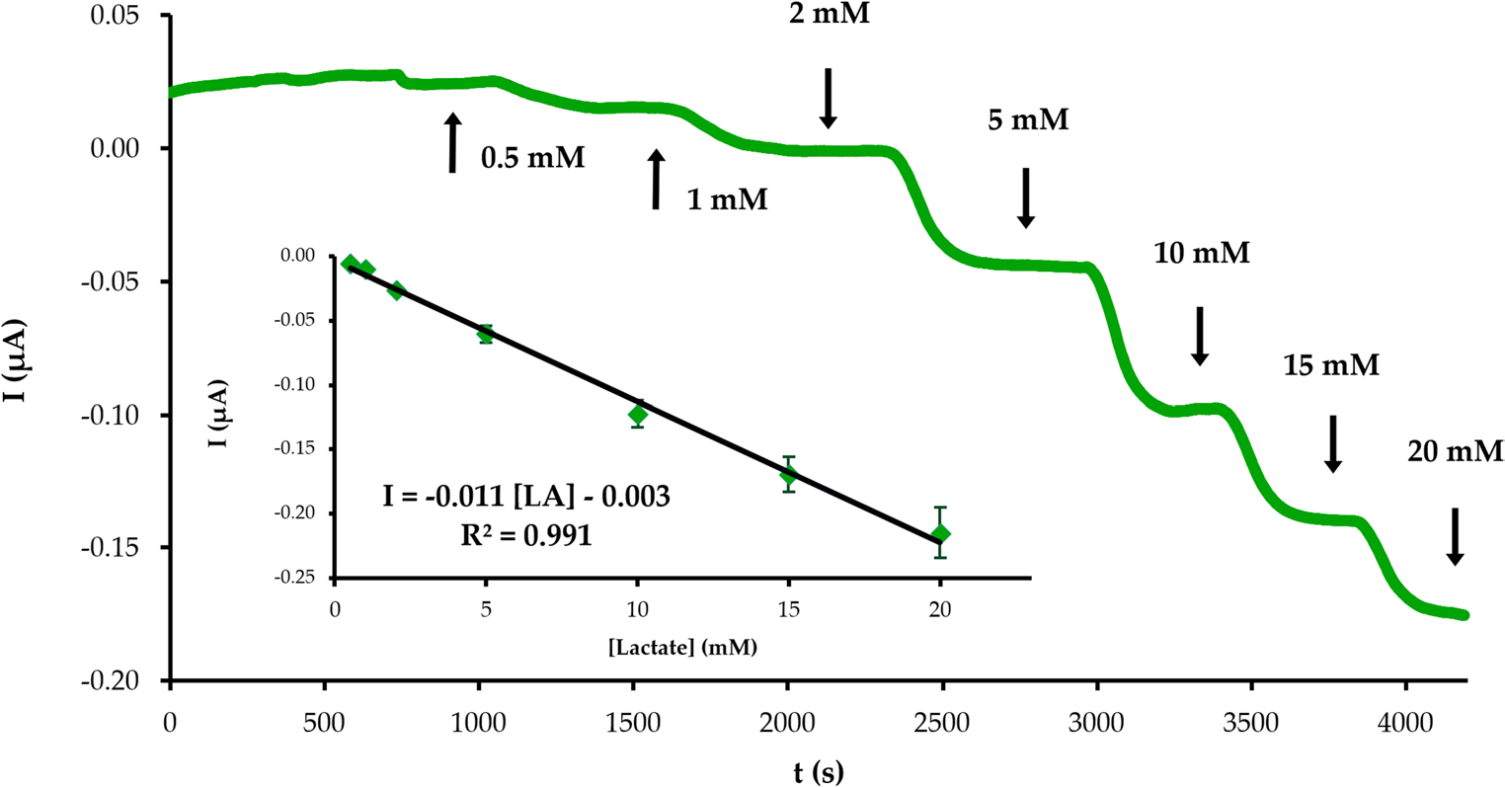
Chronoamperograms corresponding to the calibration of LA with the SNG-C-PB-LOx-AuSNPs-Chit-PVC-Nafion biosensor device in flow regime. The inset displays the calibration plot obtained. The buffer solution was 0.1 M PBS pH 7.4, the flow was 0.4 mL/min and the working potential was 0.02 V (vs. Ag-PVB-PU)

Evaluating the electroanalytical performance of the biosensor device under flow conditions is an essential step towards its applicability in lactate continuous monitoring. The calibration of LA with the developed SNG-C-based biosensor in this regime was performed using the 3D-printed electrochemical cell and the same concentration range as in batch mode. The electrode sensitivity is lower in this case due to the instability produced by the flow of the working solution, the shorter contact time between the analyte and the sensing agent and the lower amount of analyte that reaches the enzyme. Hence, the first concentration that can be accurately detected in this calibration is 0.5 mM. An excellent linear relationship (R^2^ = 0.991) was established between current intensity and LA concentrations up to 20 mM, as observed in Fig. 7. The linear regression equation (Eq. 4) obtained in this regime was (*n* = 3):

4 $$\begin{array}{l}\mathrm I\left(\mu\mathrm A\right)=-\left(0.011\pm0.002\right)\left[\mathrm{LA}\right]\\\left(\mathrm{mM}\right)-\left(0.010\pm0.007\right)\end{array}$$

The sensitivity value obtained in these calibrations was 1.06 ± 0.19 µA mM^−1^ cm^−2^ (*n* = 3). A decrease of this parameter value compared to the one obtained with the biosensor in batch mode is observed, as expected [69]. The SNG-C-PB-LOx-AuSNPs-Chit-PVC-Nafion biosensor was successfully tested for its suitability in lactate monitoring using the 3D-printed microfluidic cell device. The analysis of synthetic and real physiological samples in the flow regime would further prove this statement.

To assess the reproducibility of the microfluidic cell fabrication, the dimensions of four independently prepared cells were measured (Table S1). To evaluate the reproducibility of sensor performance within these cells, a calibration was performed for each, with the slope of the calibration curve used as the measurement parameter. Specifically, four calibrations were carried out, each in a different microfluidic cell. The results showed that the standard deviation and coefficient of variation were both, slope of calibration curve and the measurements of the microfluidic, less than 7% (*n* = 4). These findings indicate that the fabrication process for the microfluidic cells is highly reproducible.

### Analysis of synthetic, reference and real samples

Several different samples were analyzed using the SNG-C-PB-LOx-AuSNPs-Chit-PVC-Nafion biosensor device operated in flow mode to evaluate its applicability for monitoring lactate. All the samples studied were analyzed by recording the corresponding current intensity and applying the regression equation obtained in the calibration with LA. First, two synthetic samples were prepared simulating interstitial fluids, each spiked with LA concentrations of 2 µM and 4 µM, as described in Sect. 2.8. The mean recovery values obtained, defined as the ratio between the LA concentration measured using the electrochemical method and the spiked concentration, were 100% for the 2 mM LA sample and 95% for the 4 mM LA sample.

A commercial reference sample was analyzed as well to further test the applicability of the biosensor. The composition of this reference material includes all the interferents, concentrations and parameters found in blood samples and measured by the gas analyzer equipment (usually employed at hospitals). The LA concentration determined using the developed biosensor was 1.83 mM, compared to the reference value of 1.75 mM provided by the supplier. Consequently, the recovery value for the reference samples was 104%.

Finally, the proposed biosensor was applied in the flow analysis (Figure S12) of seven different untreated human serum real samples. Blood sampling was achieved in repose conditions as well as in effort conditions to examine different values of the lactate range. LA concentration in these samples was also determined using a gas analyzer and a lactate analyzer device (Lactate Scout 4, for use at home) as gold standard for comparing purposes (Table 4).

**Table 4:** Lactate values obtained by SNG-C-PB-LOx-AuSNPs-Chit-PVC-Nafion biosensor respect to the gold standard

Sample	Blood Gas analyzer	Lactate Scout analyzer	Lactate biosensor (*n* = 3)	%RSD
1 (RPS)	2.0	2.70	2.71 ± 0.05	1.75
2 (RPS)	2.2	2.20	2.27 ± 0.07	3.09
3 (E)	5.1	5.50	5.61 ± 0.11	2.57
4 (E)	5.6	8.00	8.10 ± 0.05	0.62
5 (E)	8.7	8.50	8.41 ± 0.20	2.38
6 (E)	8.3	10.18	10.31 ± 0.13	1.25
7 (E)	12	14.55	14.74 ± 0.19	1.26

*RPS* repose, *E* effort conditions

**Fig. 8:**
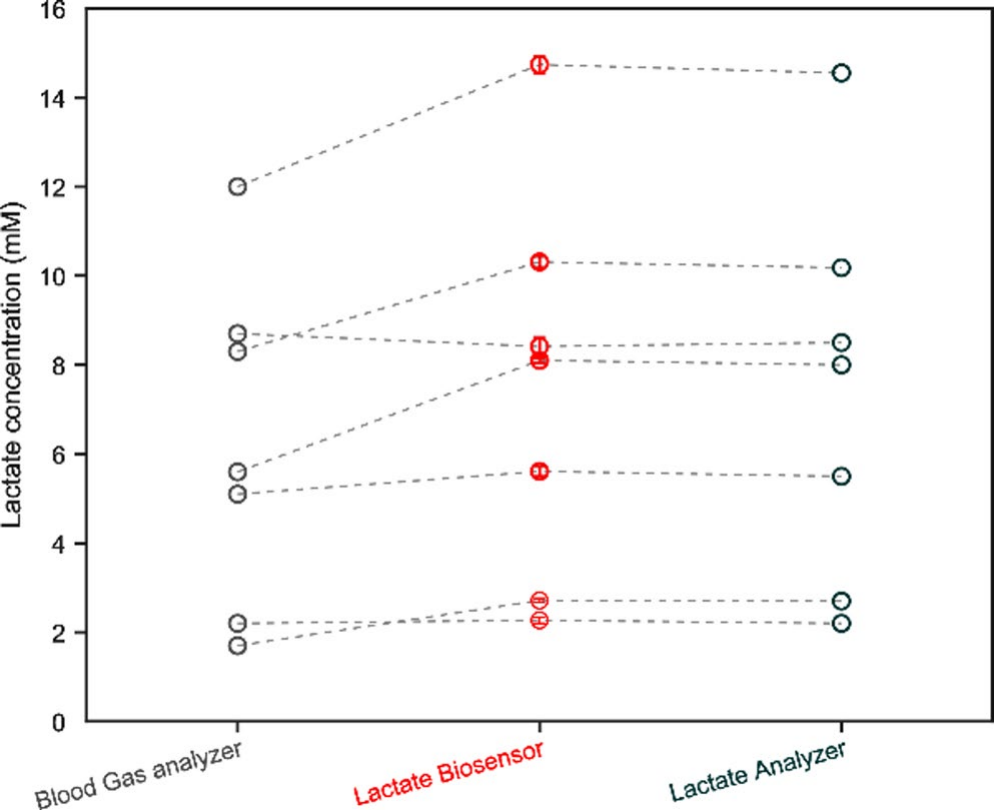
Results of the determination of lactate with the proposed biosensor and the gold standard

As it can be observed, the lactate biosensor SNG-C-PB-LOx-AuSNPs-Chit-PVC-Nafion allows for performing precise measurements for blood samples analyzed in this work, whether an effort has been made or not, with a relative standard deviation (%RSD) less than 5%. It is noteworthy to mention that all the analyses were carried out with the same biosensor in a total of 7 days and more than 16 determinations, indicating the robustness of the sensor. Concerning the accuracy of the device, it is possible to foresee some differences depending on the gold standard used as comparison. This is more evident graphically as demonstrated in Fig. 8.

Lactate concentration obtained with the Lactate Scout analyzer (for use at home) is in consonance with the concentration found with the lactate biosensor. In addition, some statistical studies were done using t-student for samples between biosensor findings and reference techniques results were accomplished. In this sense it was possible to clarify that there is no statistical evidence (p-value lower than 0.05) to suggest that the biosensor results differ significantly from the reference technique at a confidence interval of 95% The p-values obtained from these studies are collected in Table 5. This fact can also be appreciated in Figure S13 and S14. Remarkably, the errors found in these scenarios were in all cases lower than 3%, proving the excellent accuracy of the biosensor. The linear correlation between these two methods was also estimated (Eq. 5 and Figure S15B), resulting in an excellent model (R^2^ = 0.9997):

5 $$\begin{array}{l}{\lbrack\mathrm{LA}\rbrack}_{\mathrm{biosensor}}(\mathrm{mM})\\=1.0092{\lbrack LA\rbrack}_{\mathrm{Lactate}\;\mathrm{Scoutanalyzer}}(\mathrm{mM})+0.0068\end{array}$$

**Table 5:** Results of t-student sample test carried out to compare biosensors results vs. reference techniques at a confidence interval of 95%

Sample	*p*-value obtained vs. Lactate Scout	Decision*	*p*-value obtained vs. blood gas analyzer	Decision*
1	0.6734	NS	0.0007	S
2	0.2347	NS	0.2347	NS
3	0.3679	NS	0.0921	NS
4	0.2247	NS	0.0089	S
5	0.6625	NS	0.2996	NS
6	1.0000	NS	0.0285	S
7	0.3820	NS	0.0302	S

* *S* significant statistically difference, *NS* no significant statistically difference

On the contrary, for the same samples, lactate results obtained with the blood gas analyzer device, which is currently used in the surgery room, differ a bit more (Figure S15). In this case, applying the same statistical test, only 4 of the 7 results passed the test (p-value lower than 0.05), indicating statistical differences between both methodologies. The resulting values are also collected in Table 5. This can also be observed in Figure S15B. In the same manner, a linear estimation (Eq. 6 and Figure S15B) was performed, resulting in a slightly worse correlation coefficient (R^2^ = 0.9403):

6 $$\begin{array}{l}{\lbrack\mathrm{LA}\rbrack}_{\mathrm{biosensor}}\;(\mathrm{mM})\\=1.1491\;{\lbrack\mathrm{LA}\rbrack}_{\mathrm{blood}\;\mathrm{gas}\;\mathrm{analyzer}}\;(\mathrm{mM})+0.2937\end{array}$$

Particularly, due to previous work [30] authors have noticed less precision in the results of this gold standard. Moreover, the blood gas analyzer is designed to measure whole blood directly without centrifugation, whereas the samples analyzed in this work were subjected to a brief centrifugation step after collection. This difference in sample handling may have affected the lactate concentrations measured by the proposed biosensor and the blood gas analyzer, partially explaining the discrepancy observed in Fig. 8. In spite of the discrepancies observed, the deviations of the lactate biosensor with respect to both reference techniques did not exceed 20%. According to bibliography [98], such a margin could be considered acceptable. Nevertheless, this is beyond the scope of the present piece of research. However, with the results obtained in this work and the validation step performed, it should be noted that the proposed biosensor provides precise and accurate lactate concentration results in serum samples. This demonstrates that the enzyme works perfectly, in a blood-like matrix, even at high lactate concentrations, using a continuous regime and a 3D printed microfluidic chamber.

## Conclusions

In this work, a multipolymeric layer biosensor has been manufactured and successfully applied in the electroanalysis of LA. Affordable SNG-C electrodes were used as a base for the fabrication of this sensing device. This biosensor exhibited adequate linear response in the concentration range from 0.2 to 20 mM, thanks to the deposition of a diffusion membrane and has been proposed as a suitable approach for lactate monitoring in normal conditions or under extreme exercise. Compared to existing approaches, this system introduces a novel combination of materials and design, improving performance under complex conditions. The figures of merit obtained in the analytical calibration of LA in batch mode made this biosensor competitive with other PB-based devices reported in the literature. A 3D-printed microfluidic cell was designed and fabricated for the application of this biosensor in a flow regime. This device possesses several features, such as fast preparation, eco-friendly character and low cost (1.5 €). Importantly, the design of this microfluidic cell allows for potential adaptation to detect other biomarkers, including pH, Na^+^, K^+^, and glucose, and future studies will focus on modifying it for simultaneous multi-analyte detection. The feasibility of the application of this biosensor in continuous LA monitoring was assessed using this lab-made microfluidic cell. The electroanalysis of human serum samples was carried out using this tandem, demonstrating its applicability in complex biological matrices. The metabolite levels obtained were compared with the values provided by other analytical techniques, obtaining meaningful similarities between them. These results indicate that the tandem developed (SNG-C-based biosensor and 3D-printed microfluidic cell) is a promising alternative for the monitoring of LA in the field of medicine. Notably, the system ability to directly analyze untreated serum samples highlights its potential for real-world clinical applications, where simplicity and robustness are paramount.

## Supplementary Information

Below is the link to the electronic supplementary material.

Supplementary Material 1

## Data Availability

The data used to support the findings of this study are available from the corresponding authors upon request.
