# Supplementary material for: Advancement in continuous lactate determination in untreated human serum: multipolymer-based amperometric biosensor coupled with a low-cost 3D-printed microfluidic cell
Source: Mikrochim Acta. 2025 Dec 27;193(1):48. doi: 10.1007/s00604-025-07771-0 (PMC12743661; doi:10.1007/s00604-025-07771-0)
Supplement: Supplementary file 1 — Supplementary Material 1 [file 604_2025_7771_MOESM1_ESM.docx]

**Supplementary Material**

Advancement in continuous lactate determination in untreated human serum: Multipolymer-based amperometric biosensor coupled with a low-cost 3D-printed microfluidic cell

Álvaro Jesús Sainz-Calvo^1†^, Alfonso Sierra-Padilla^1†^, Dolores Bellido-Milla^1^, Lorena Blanco-Díaz^1^, Juan Jesús Fernández-Alba^2^, Carmen González-Macías^2^, Juan José García-Guzmán^1,*^, José María Palacios-Santander^1,*^ and Laura Cubillana-Aguilera^1^

^1^Institute of Research on Electron Microscopy and Materials (IMEYMAT), Department of Analytical Chemistry, Faculty of Sciences, Campus de Excelencia Internacional del Mar (CEIMAR), University of Cadiz, Campus Universitario de Puerto Real, Polígono del Río San Pedro S/N, Puerto Real, 11510 Cádiz, Spain;

^2^Departamento de Obstetricia y Ginecología, Hospital Universitario de Puerto Real, 11510 Puerto Real, Cádiz, Spain

[alvarojesus.sainz@uca.es](mailto:alvarojesus.sainz@uca.es) (A.J.S.-C); [alfonso.sierra@uca.es](mailto:alfonso.sierra@uca.es) (A.S.-P); juanjo.garciaguzman@uca.es (J.J.G.-G.); lorena.blancodiaz@alum.uca.es (L.B.-D.); dolores.milla@uca.es (D.B.-M.); [jjesus.fernandez@uca.es](mailto:jjesus.fernandez@uca.es); (J.J.F.-A); [mcarmen.gonzalez@uca.es](mailto:mcarmen.gonzalez@uca.es) (C.G.-M); laura.cubillana@uca.es (L.C.-A.); [josem.palacios@uca.es](mailto:josem.palacios@uca.es) (J.M.P.-S)

*****Correspondence: [josem.palacios@uca.es](mailto:josem.palacios@uca.es) (J.M.P.-S); [juanjo.garciaguzman@uca.es](mailto:dolores.milla@uca.es) (J.J.G.-G.)

†These authors equally contributed to the work.

**Supplementary Tables** **S2**

**Supplementary Figures**  **S3**

**Supplementary Tables**

Table S1. Dimensions of the upper and lower sections of several (n=4) microfluidic cells.

| 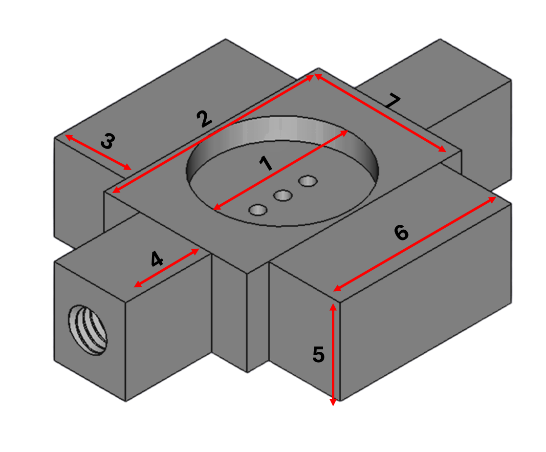  **Lower part of the microfluidic cell** | **Number part**: Dimension ± SD (n=4) | **%CV** |
| --- | --- | --- |
|  | **1:** 19.57 ± 0.45 mm | 2.29 |
|  | **2:** 30.03 ± 0.06 mm | 0.19 |
|  | **3:** 10.83 ± 0.85 mm | 7.21 |
|  | **4:** 13.07 ± 0.68 mm | 5.25 |
|  | **5:** 11.77 ± 0.7 mm | 5.95 |
|  | **6:** 20.10 ± 0.10 mm | 0.49 |
|  | **7:** 20.27 ± 0.29 mm | 1.43 |
| 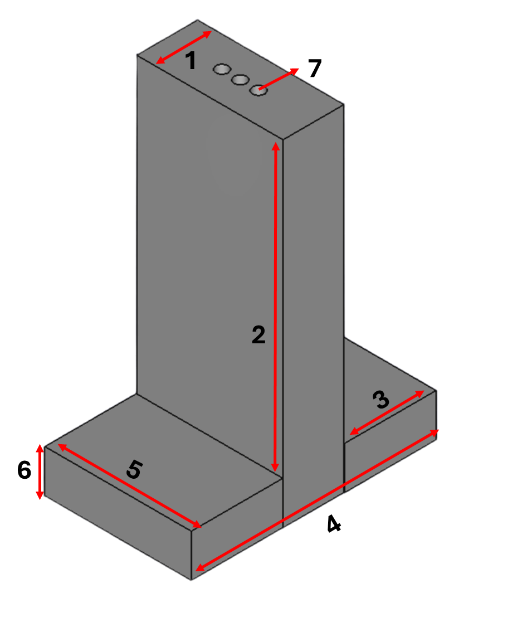  **Upper part of the microfluidic cell** | **Number part**: Dimension ± SD (n=4) | **%CV** |
|  | **1:** 9.93 ± 0.12 mm | 1.21 |
|  | **2:** 40.63 ± 0.25 mm | 0.62 |
|  | **3:** 15.40 ± 0.53 mm | 3.44 |
|  | **4:** 40.27 ± 0.23 mm | 0.57 |
|  | **5:** 24.90 ± 1.10 mm | 4.42 |
|  | **6:** 6.87 ± 0.42 mm | 6.11 |
|  | **7:** 1,97 ± 0.06 | 3.05 |

**Supplementary Figures**

***
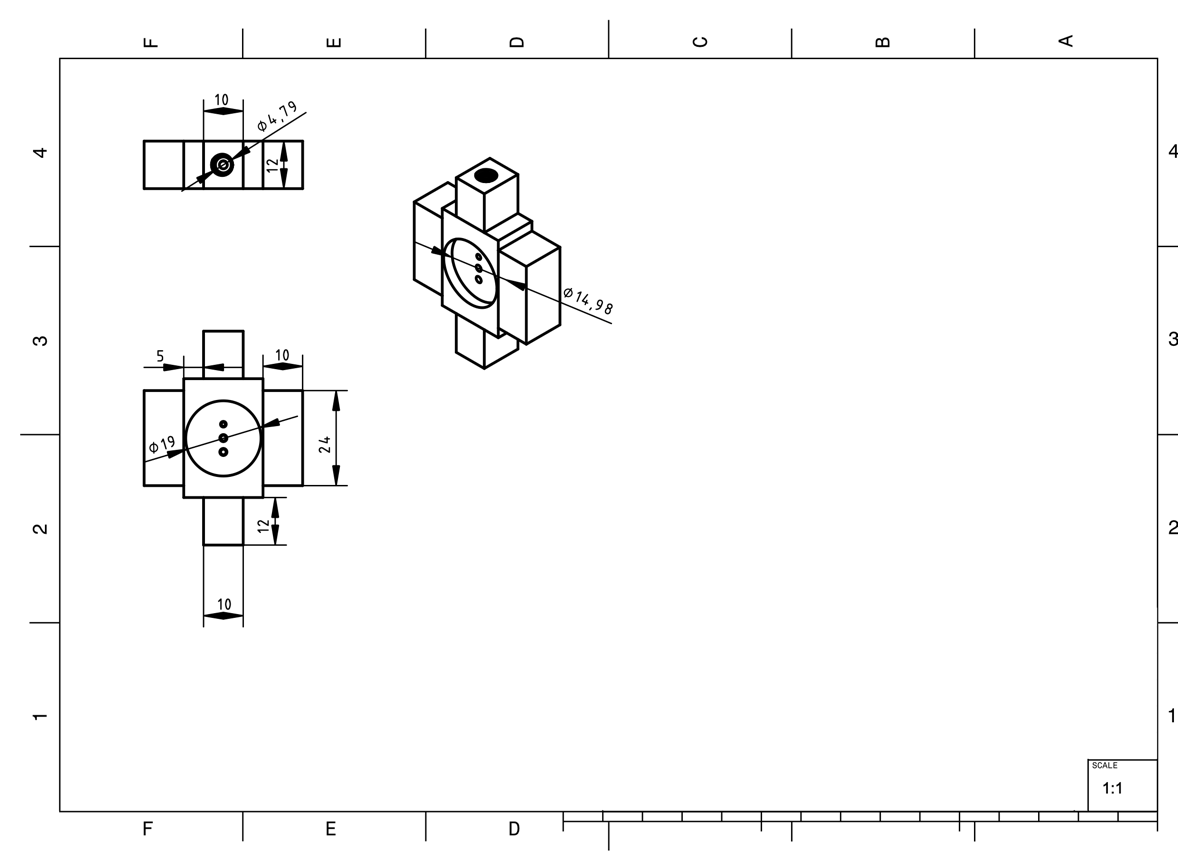
***

**Figure S1.** Representation of the lower part of the 3D-printed microfluidic cell for Sonogel-Carbon electrodes**.**

***
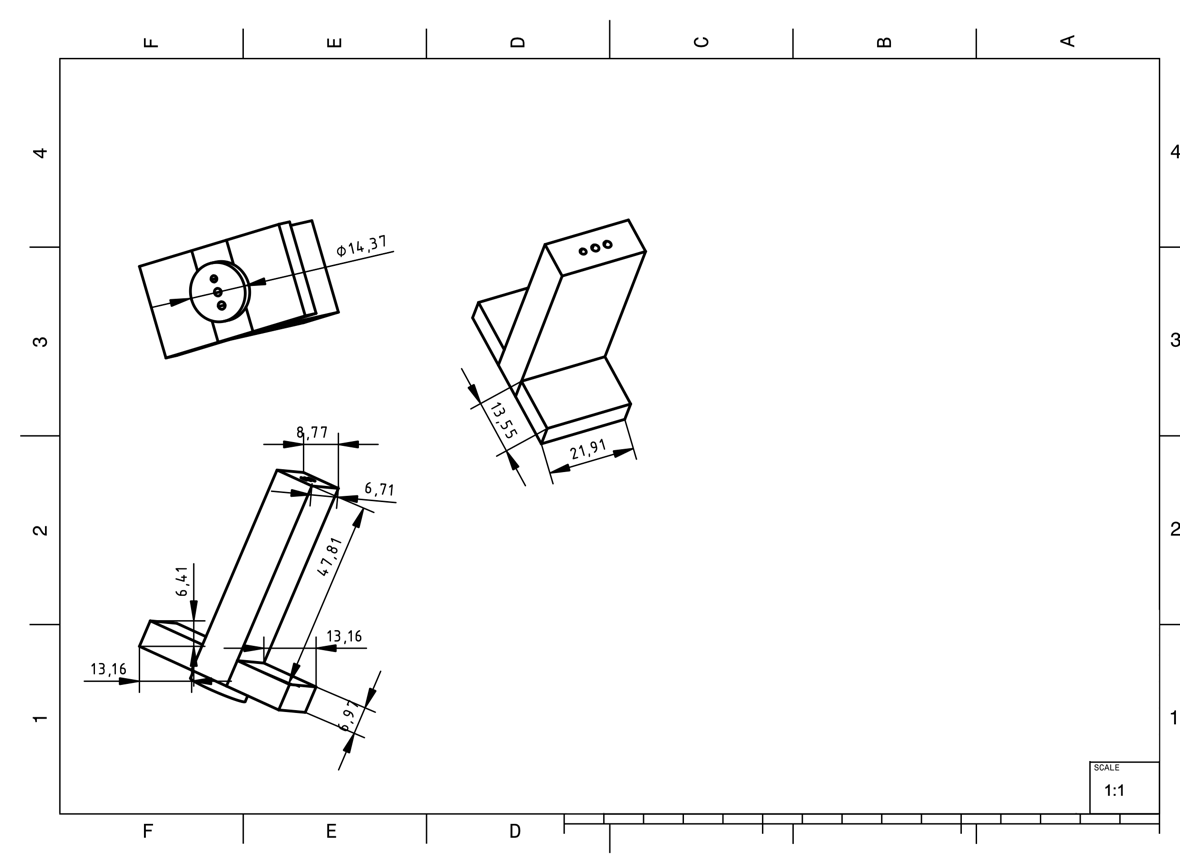
***

**Figure S2.** Representation of the upper part of the 3D-printed microfluidic cell for Sonogel-Carbon electrodes**.**


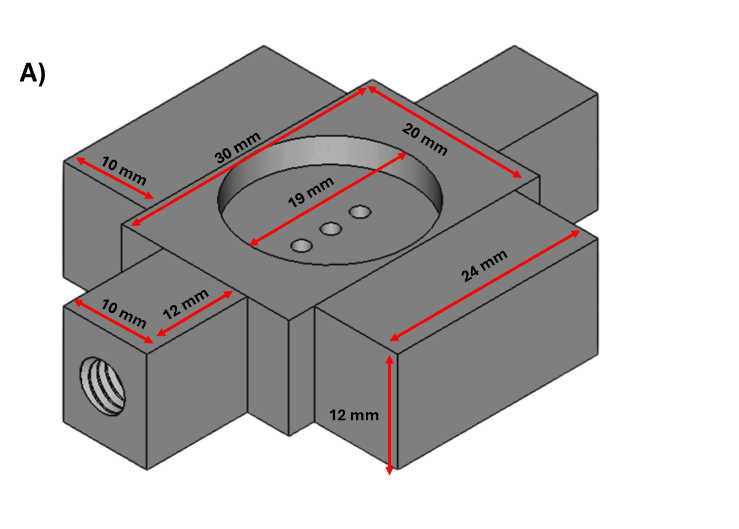


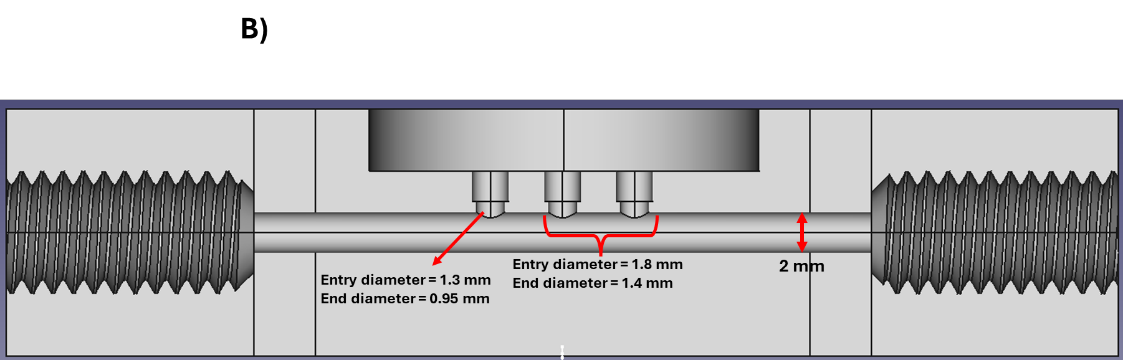


**Figure S3**. Dimensions of the lower part of the microfluidic chamber. (A) Full view of the bottom part of the chamber, showing the overall layout and external dimensions and (B) detail of the microfluidic cell viewed from a cross-sectional plane along the X-axis, highlighting the internal dimensions relevant to fluid flow.


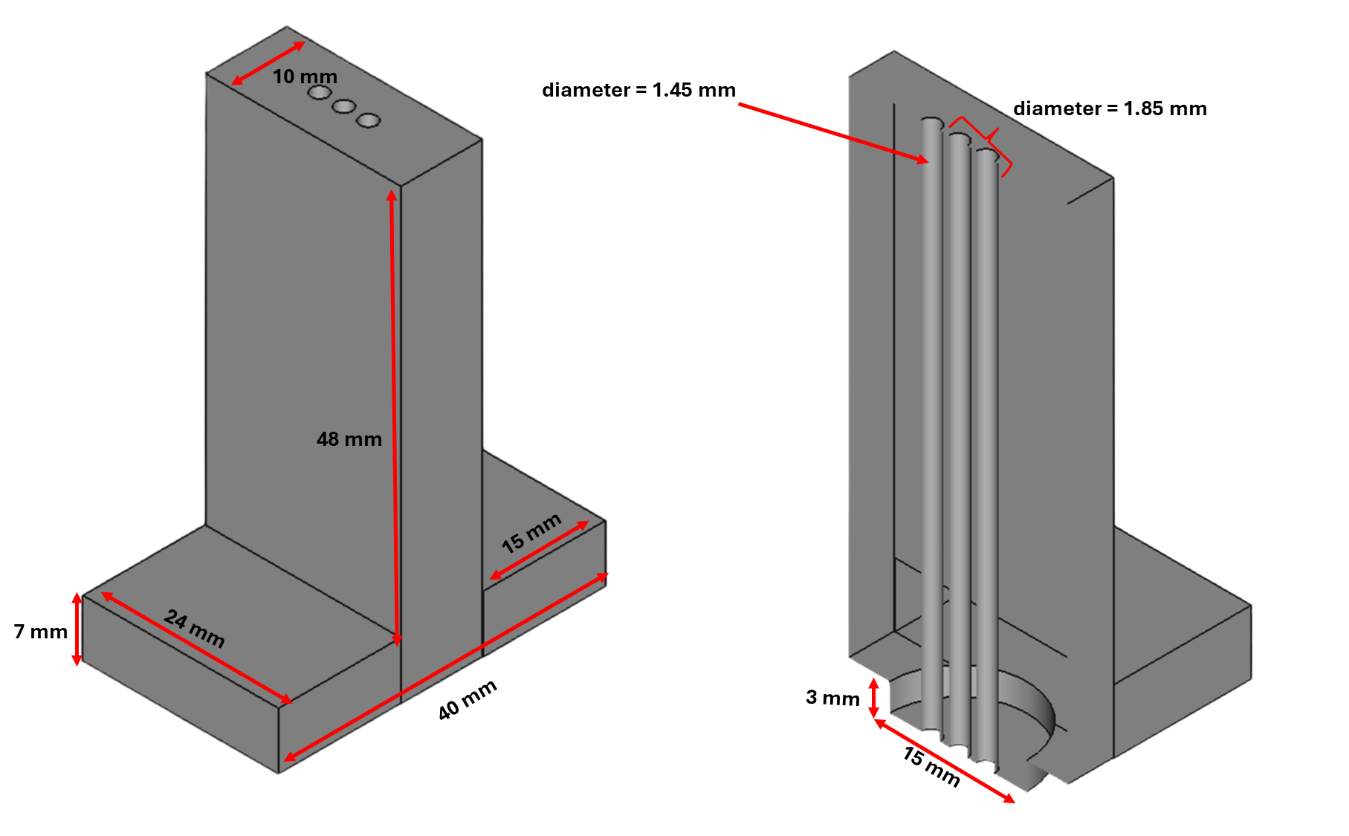


**Figure S4.** Dimensions of the upper part of the microfluidic chamber, illustrating the layout and key features relevant to fluid inlet, outlet, and sealing.


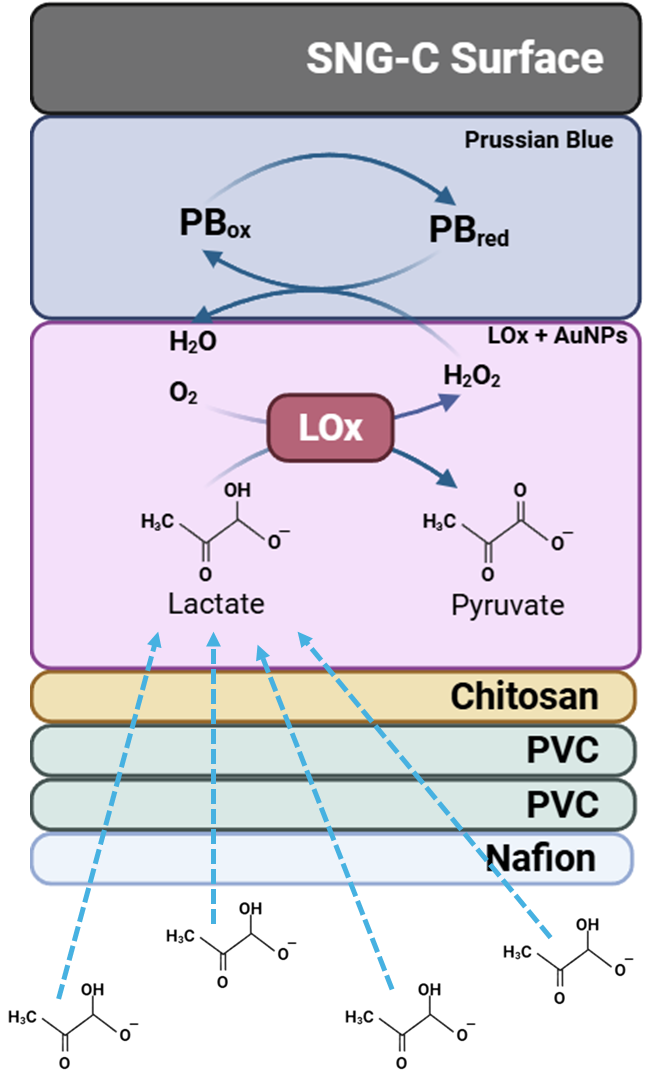


**Figure S5**. Schematic representation of the operating principle of the lactate amperometric biosensor based on the SNG-C-PB-LOx-AuSNPs-Chit-PVC-Nafion electrode.

**Figure S6.** (A) UV-Vis spectrum of the gold nanoparticles synthetized using high-energy ultrasound. (B) Size distribution of the gold sononanoparticles obtained with the dynamic light scattering technique.


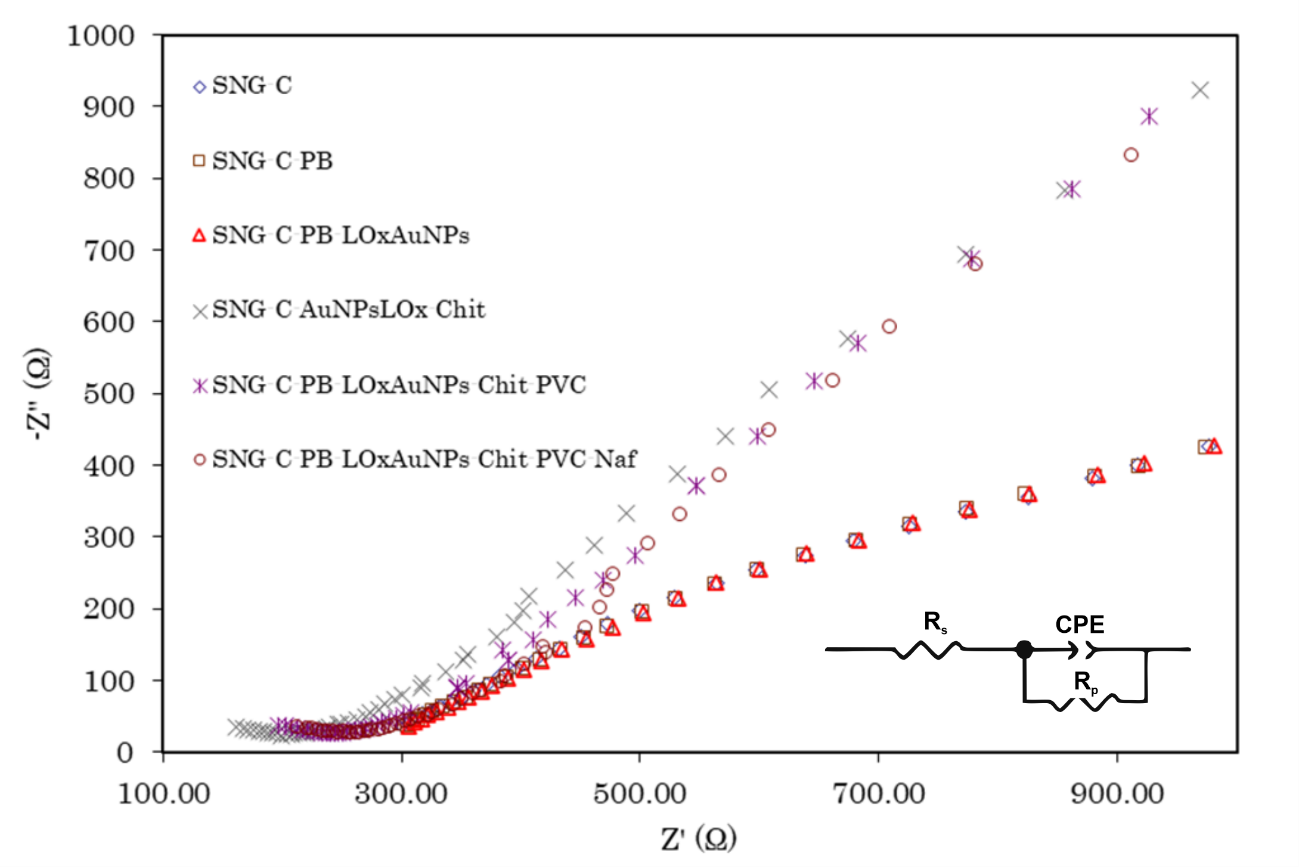


**Figure S7**. Nyquist diagrams of SNG-C, SNG-C-PB, SNG-C-PB-LOxAuNPs, SNG-C-PB-LOxAuNPs-Chit, SNG-C-PB-LOxAuNPs-Chit-PVC and SNG-C-PB-LOxAuNPs-Chit-PVC-Nafion electrodes. Frequency range: 10 kHz–100 mHz in 0.5 mol L⁻¹ KCl solution containing 5 mmol L⁻¹ Fe(CN)₆³⁻/⁴⁻. The inset represents the equivalent circuit.

***
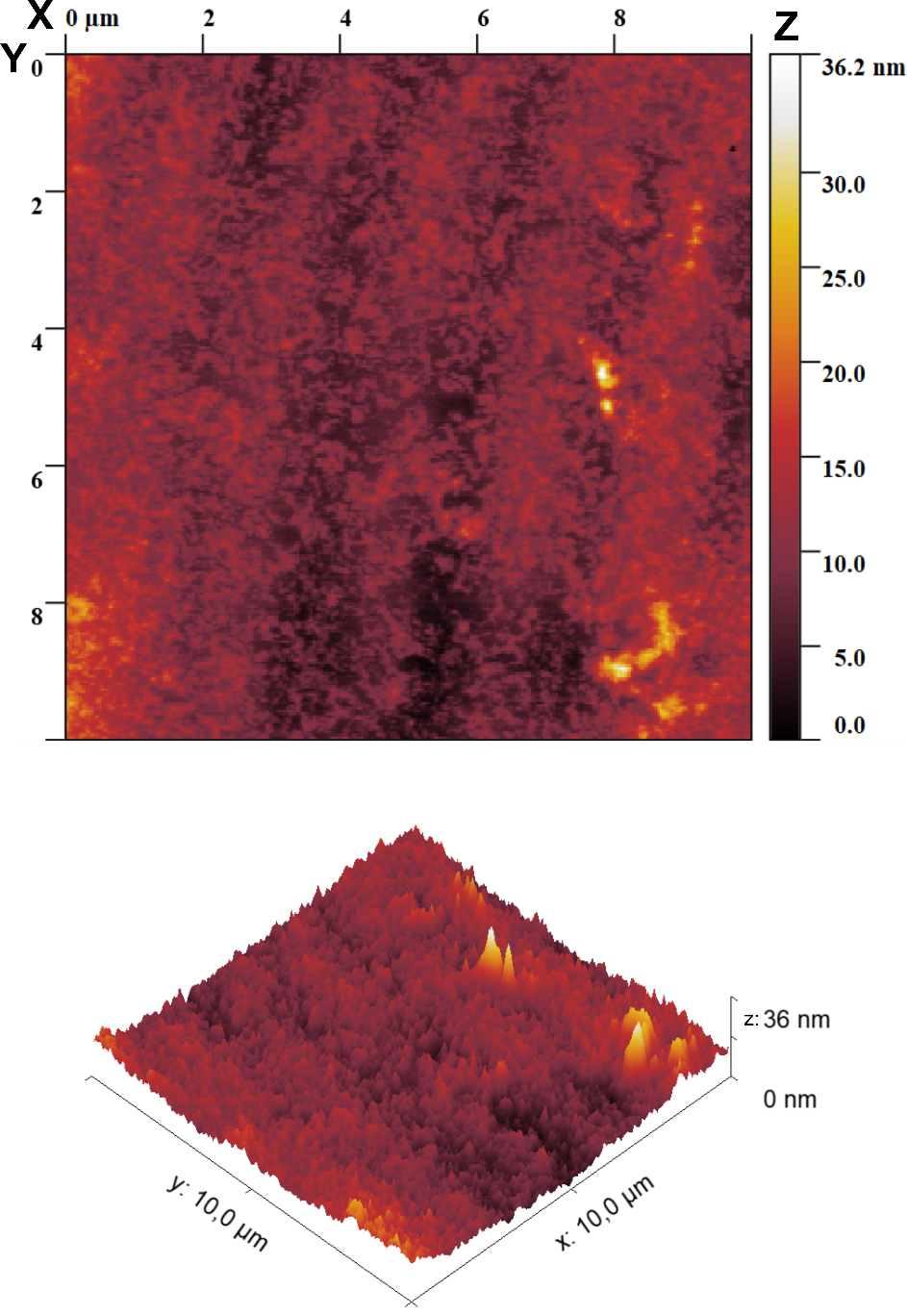
***

**Figure S8**. AFM micrograph of the SNG-C-PB-LOx-AuSNPs-Chit-PVC-Nafion biosensor surface in tapping mode (scan area = 100 µm^2^).


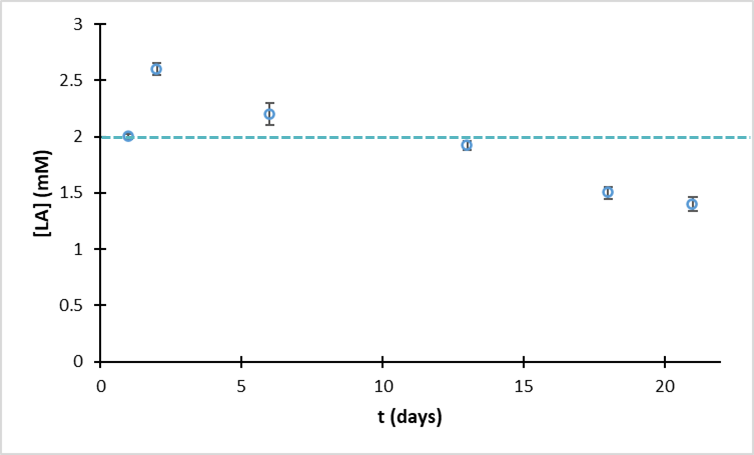


**Figure S9**. Study of the lactate biosensor (SNG-C-PB-LOx-AuSNPs-Chit-PVC-Nafion) lifetime by recording the signal at a theoretical lactate concentration of 2 mM.


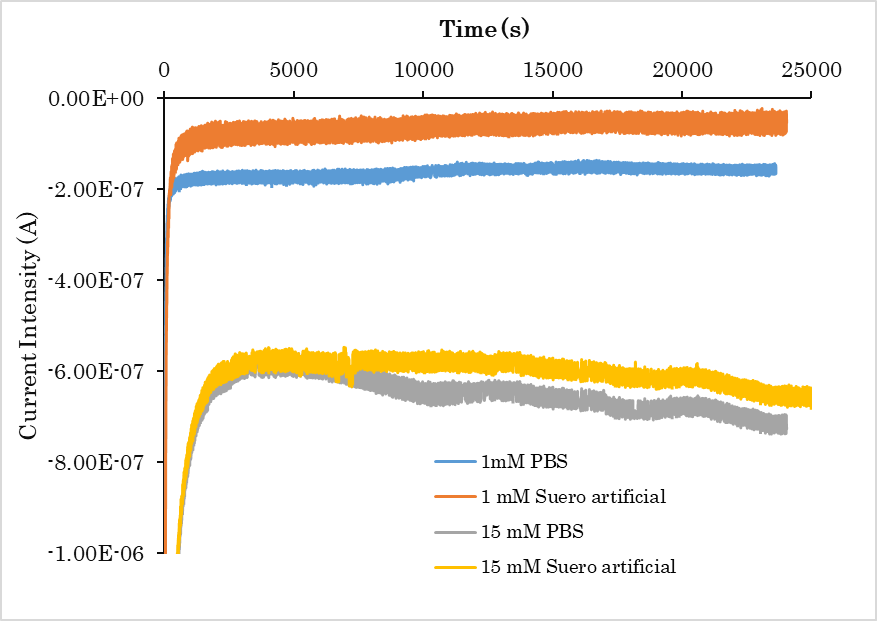


**Figure S10**. Chronoamperogram of the long-term response of the SNG-C-PB-LOx-AuSNPs-Chit-PVC-Nafion biosensor device toward lactate at different concentrations and media. The measurements were performed at 1 mM lactate in PBS (blue) and artificial serum (red), as well as at 15 mM lactate in PBS (green) and artificial serum (purple). The applied potential was 0.1 V (vs. Ag/AgCl).

**Figure S11.** Cyclic voltammograms recorded in 5 mM [Fe(CN)_6_]^-3^ and 0.5 M KCl solution with a SNG-C electrode using as reference electrodes: a commercial Ag/AgCl 3M KCl electrode (blue line) or a modified Ag rod with PVB and PU (red line). Scan rate was 50 mV/s.


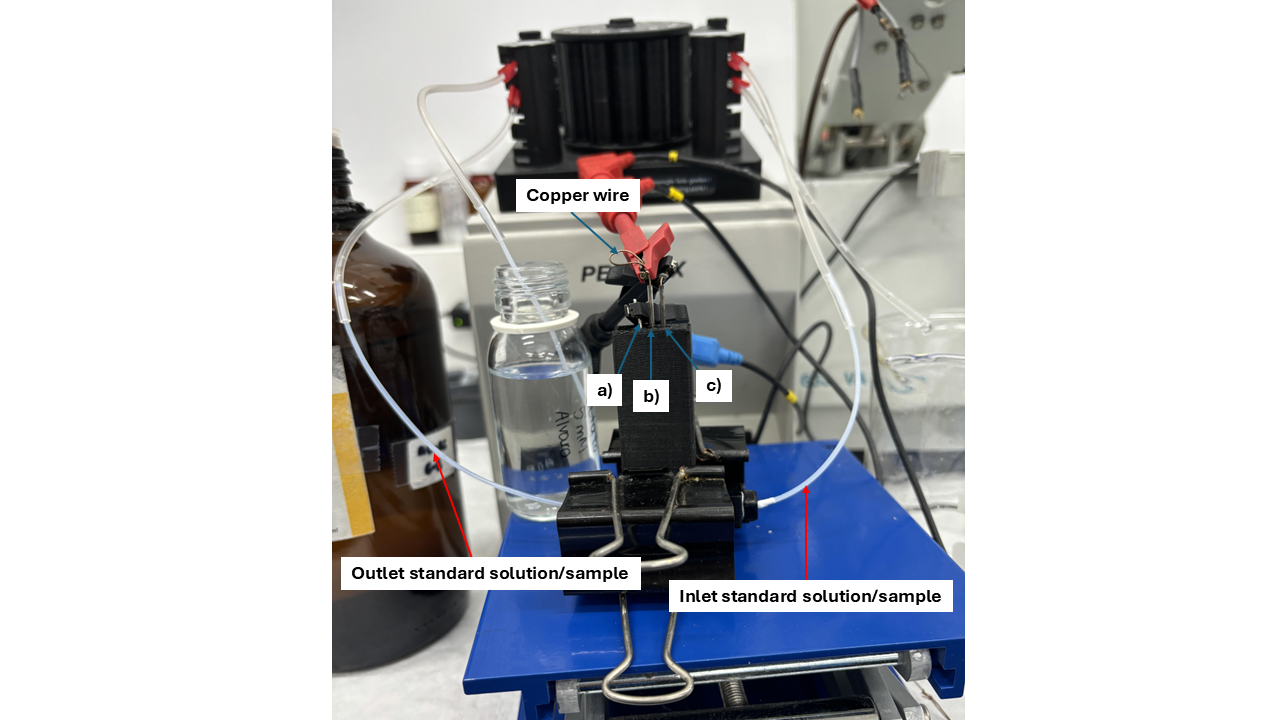


**Figure S12.** Diagram of the measurement system in continuous mode. (A) Pseudo-reference electrode prepared in the laboratory; (B) SNG-C-PB-LOxAuNPs-Chit-PVC-Nafion biosensor (working electrode); and (C) SNG-C electrode (auxiliary electrode).

***
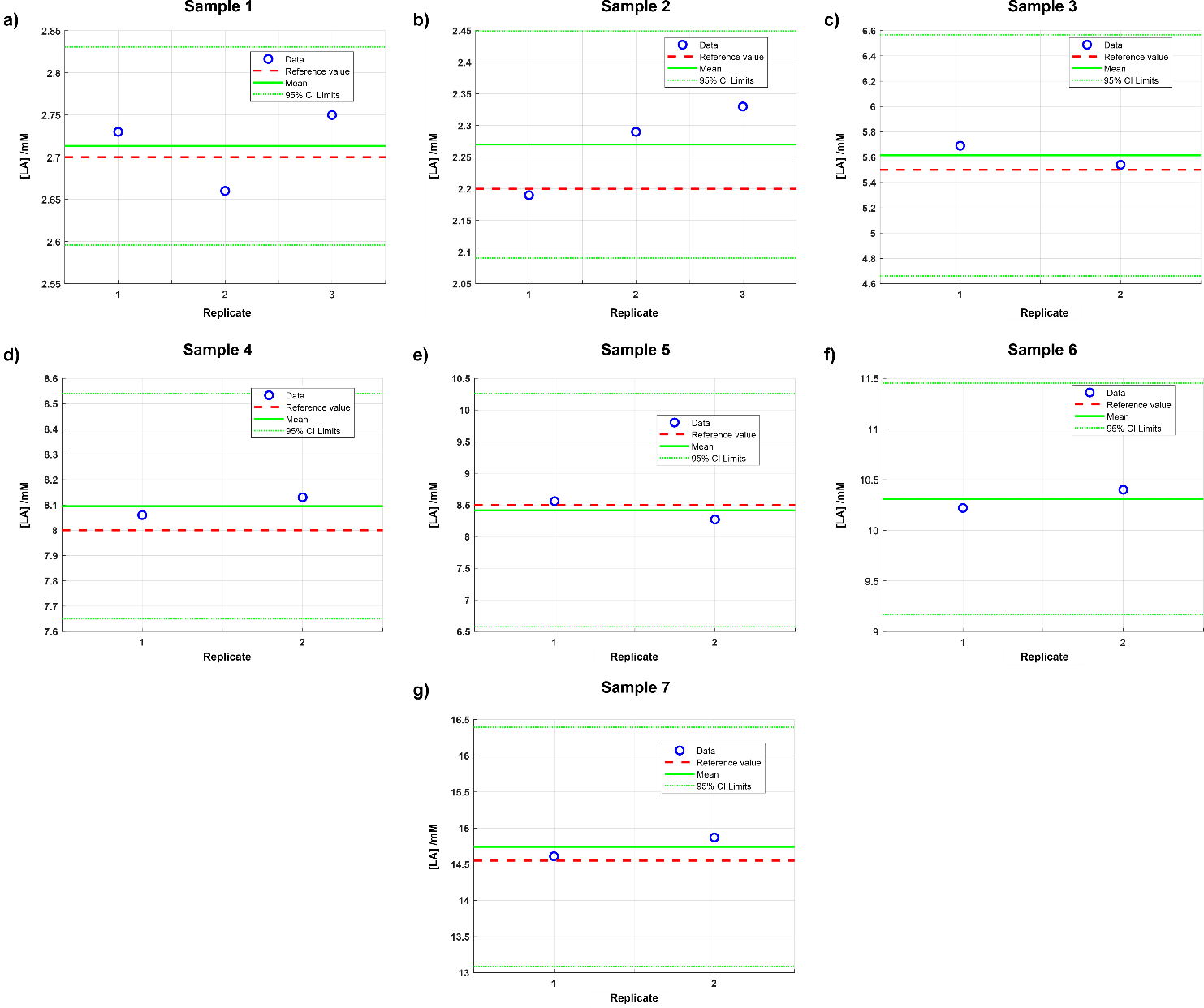
***

**Figure S13.** Statistical analysis of the results of the biosensor in comparison with the Lactate Analyzer gold technique: (a) repose sample 1; (b) repose sample 2; (c) medium effort sample 3, (d) medium effort sample 4, (e) medium effort sample 5, (f) medium effort sample 6 and (g) high effort sample 7.

***
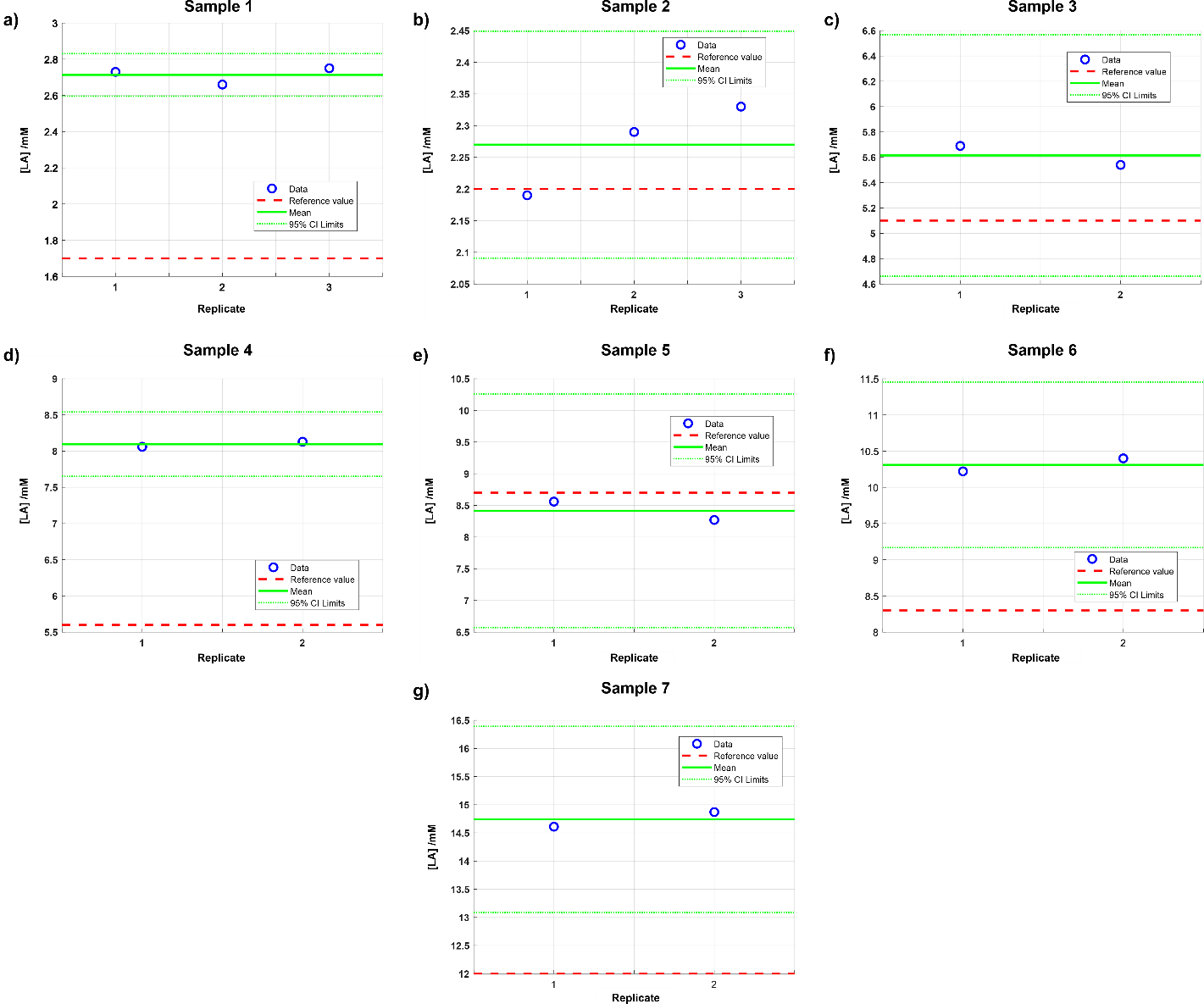
***

**Figure S14.** Statistical analysis of the results of the biosensor in comparison with the Blood gas Analyzer gold technique: (a) repose sample 1; (b) repose sample 2; (c) medium effort sample 3, (d) medium effort sample 4, (e) medium effort sample 5, (f) medium effort sample 6 and (g) high effort sample 7.


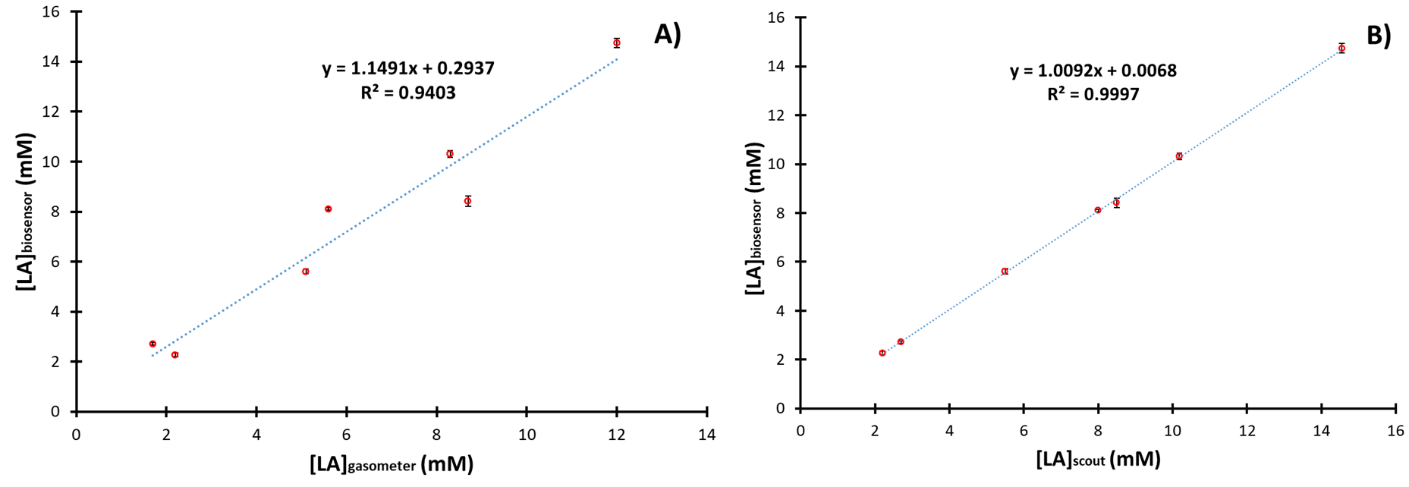


**Figure S15**. Comparison of lactate concentrations obtained using the biosensor SNG-C-PB-LOx-Chit-PVC-Naf versus those measured with the (A) Gas analyser usually employed at hospitals and (B) lactate Scout analyser
